# A comprehensive characterization of opsin distribution across the visual system in the three castes of the honeybee

**DOI:** 10.1016/j.isci.2025.113105

**Published:** 2025-07-16

**Authors:** Alexandre Durand, Sarah Larnaudie, Dorian Champelovier, Brice Ronsin, Isabelle Lafon, Martin Giurfa

**Affiliations:** 1 Research Centre on Animal Cognition, Centre of Integrative Biology, CNRS, University Paul Sabatier, F-31062 Toulouse, France; 2 Centre of Integrative Biology, CNRS, University Paul Sabatier, F-31062 Toulouse, France; 3 Sorbonne Université, CNRS, Inserm, Center of Neuroscience Neuro-SU, F-75005 Paris, France; 4 Sorbonne Université, CNRS, Inserm, Institut de Biologie Paris-Seine, IBPS, F75005 Paris, France

**Keywords:** entomology, evolutionary biology, zoology

## Abstract

Honeybees see the world through photoreceptors in their compound eyes and ocelli, which express four opsins: UVOP (UV-sensitive), BLOP (blue-sensitive), and LOP1/LOP2 (both green-sensitive). Here, we provide the first comparative mapping of opsin RNA across the three bee castes: workers, drones, and queens. In all castes, *Lop1* was exclusive to the compound eyes, while *Lop2* was confined to the ocelli, which also contained *Uvop* photoreceptors. Queens and workers exhibited three ommatidial types in their compound eyes, with six *Lop1* photoreceptors, alongside either one *Uvop* and one *Blop*, two *Uvop* or two *Blop* photoreceptors. Drones shared the same ommatidial types ventrally, but the upper two-thirds of their compound eyes exhibited only two ommatidial types: one with one *Uvop* and seven *Blop* photoreceptors, the other with two *Uvop* and six *Blop* photoreceptors. Our findings reveal unknown caste-specific adaptations in bee vision, enhancing understanding of the evolutionary diversity of insect visual systems.

## Highlights

• Honeybees have 4 opsin types: Uvop, Blop, Lop1, and Lop2
• The ocelli of workers, queens and drones present Uvop and Lop2 cells
• Worker and queen ommatidia have 6 Lop1 and either Uvop+Blop, 2 Uvop, or 2 Blop cells
• Drone ommatidia have abundant Blop cells dorsally and a female-like pattern ventrally

## Graphical abstract

**Figure:**
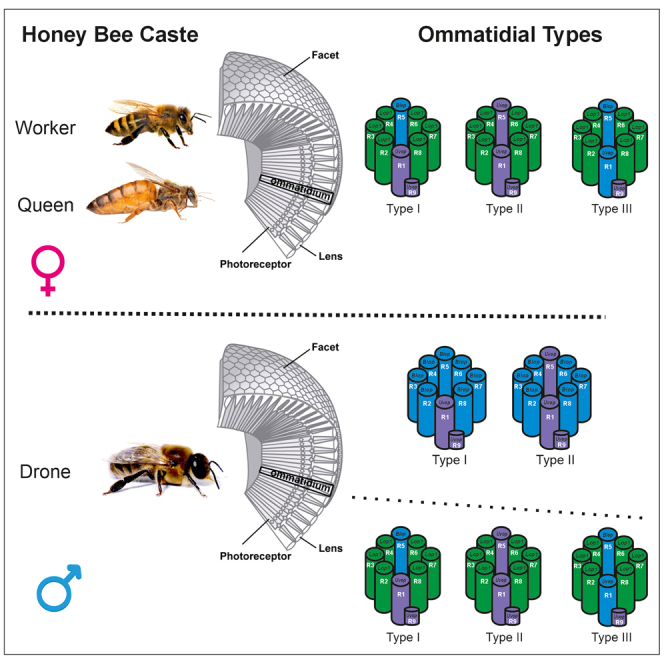

## Introduction

Color vision is the ability to distinguish surfaces based on their chromatic content, independent of differences in brightness.^1^ This capacity is directly related to the presence and function of retinal photoreceptors with different chromatic sensitivities, which respond to specific wavelength ranges. These color photoreceptors provide input to color-opponent neurons in the central nervous system, where the perception of color arises.^2^^,^^3^^,^^4^

The domestic honeybee, *Apis mellifera*, has been a key model for studying color vision.^5^ Since Karl von Frisch’s pioneering work, which demonstrated bees’ ability to see colors and characterized their color-vision spectrum (from 300 nm to 650 nm),^6^^,^^7^ much has been learned about the behavioral and physiological bases of bee color vision.^5^^,^^8^^,^^9^^,^^10^^,^^11^ Honeybees view the world through compound eyes, made up of functional units called ommatidia. Workers and queens have an average of 5,300 and 4,600 ommatidia, respectively, while drones, which rely heavily on vision for spotting queens against the sky during mating flights, have about 9,900.^12^

Each ommatidium consists of a corneal lens, a crystalline cone (CC), and photoreceptor cells surrounded by peripheral pigment cells. In honeybee workers, each ommatidium contains nine photoreceptor cells arranged concentrically and twisted along the ommatidial axis,^13^ except in the dorsal rim area (DRA) of the compound eye, a region specialized for detecting the polarization of skylight for navigation purposes.^14^ The inner part of each photoreceptor cell extends a series of membrane protrusions called microvilli toward the center of the ommatidium, forming the rhabdom—a central photoreceptive structure where the microvilli from all photoreceptors converge. Microvilli contain rhodopsin molecules, which absorb photons of light and trigger the biochemical events leading to the generation of an electrical signal—a process known as phototransduction.^15^

Rhodopsins are composed of retinal, a chromophore that captures light, and opsins, which are seven-transmembrane-domain proteins belonging to the G protein-coupled receptor (GPCR) superfamily.^16^ Structural differences between opsins create varying sensitivities to light at different wavelengths, forming the basis for distinct spectral sensitivities. Honeybees have three types of photoreceptors in the retina of their compound eyes, each expressing a different opsin type. Electrophysiological studies on worker bees have characterized the spectral sensitivity of these photoreceptor classes^17^^,^^18^: one is maximally sensitive in the ultraviolet (UV) region of the spectrum (350 nm, UV or S type), another in the blue region (440 nm, blue or M type) and the third one is maximally sensitive in the green region of the spectrum (540 nm, green or L type). The input from these receptors is combined in an antagonistic manner in color-opponent neurons in the brain,^19^^,^^20^ creating the basis for color perception.

*In-situ* hybridization studies on worker bee ommatidia have revealed the presence and distribution of opsin types in their compound eyes.^13^ The UV-sensitive opsin was named *UVOP*,*^21^* the blue-sensitive opsin *BLOP*,*^21^* and the green-sensitive opsin, *LOP*.^22^ Three classes of ommatidia were identified: all contain six green-sensitive photoreceptors expressing *Lop* RNA, which have short visual fibers (svf) terminating in the lamina (R2-R4 and R6-R8 photoreceptors).^23^ One class also includes two UV-sensitive photoreceptors (expressing *Uvop* RNA), a second contains two blue-sensitive photoreceptors (expressing *Blop* RNA), and the third includes one UV-sensitive and one blue-sensitive photoreceptor.^13^ These two photoreceptors (R1 and R5 photoreceptors), varying in opsin expression, have long visual fibers (lvf) projecting deeper to the medulla.^18^^,^^23^ The three ommatidial classes are randomly distributed across the compound eye, with a concentration of blue receptors in the ventral part of the eye and UV receptors dominating the DRA. This specialized region, which points toward the sky, helps bees detect polarized light for navigation.^24^

A second green opsin, named LOP2, was later identified in the bee genome, prompting the renaming of the previously known green opsin to LOP1.^25^ *In-situ* hybridization analyses were conducted for LOP1 and LOP2 in worker bees, although no quantitative data on opsin distribution within individual ommatidia was provided.^25^ This study described the regional distribution of these opsins in both the compound eyes and ocelli, the simple eyes composed of a single lens and various underlying photoreceptors that do not build an elaborate retina^26^ and primarily respond to light intensity for orientation and flight. Honeybees have one medial and two lateral ocelli located at the top of their head. Electrophysiological studies identified two photoreceptor types in the ocelli, one absorbing light at around 340 nm and the other at around 500 nm.^27^^,^^28^ Velarde et al.'s *in-situ* hybridization analyses^25^ confirmed the presence of both *Uvop* and *Lop2* RNA in the ocelli, concluding that *Lop1* is specific to the compound eyes while *Lop2* is specific to the ocelli. The same study also mapped the distribution of *Uvop*, *Blop*, and *Lop1* across the compound eyes of drones,^25^ though no detailed analysis of ommatidial classes or their distribution was performed. A later study using qPCR quantified opsin mRNA levels in the compound eyes of workers and drones from larval to adult stages but similarly did not analyze ommatidial classes.^29^ Remarkably, no research has yet examined opsin distribution in the visual system of queens, likely due to the challenge of removing the sole queen from a colony and the difficulty in obtaining robust sample sizes.

Differences in opsin distribution could be expected because queens spend most of their life in the darkness of the hive and restrict their experience of the external world to the short mating flight. On the contrary, worker bees perform foraging tasks, which translate into hundreds of foraging flights in natural environments. The fact that queens have less ommatidia than workers is consistent with this difference in life styles. In addition, drones are also expected to differ from workers, as their larger number of ommatidia is associated with a prominent upwards-facing acute zone characterized by larger facet diameters, smaller inter-ommatidial angles, and larger rhabdoms.^30^^,^^31^ This specialization reflects their reliance on vision and the need for higher spatial acuity to detect queens against the sky during the nuptial flight.

Here, we present the first comprehensive characterization of opsin distribution in the compound eyes and ocelli of the three honeybee castes: workers, drones, and queens. Using fluorescence *in situ* hybridization (FISH), we analyzed the expression of *Uvop*, *Blop*, *Lop1*, and *Lop2* RNAs within the ommatidia of both the compound eyes and ocelli in each caste. By mapping opsin distribution across the compound eye and ocelli, as well as within individual ommatidia, we provide new insights into the bee visual system and the caste-specific differences that emerge. We discuss our findings from a comparative perspective, focusing on the visual systems of flies, butterflies, and other insects. These results generate new hypotheses for future functional analyses of visual capabilities in honeybees and for a better understanding of the evolutionary diversity of insect visual systems.

## Results

### Validation of the FISH technique

**Figure 1:**
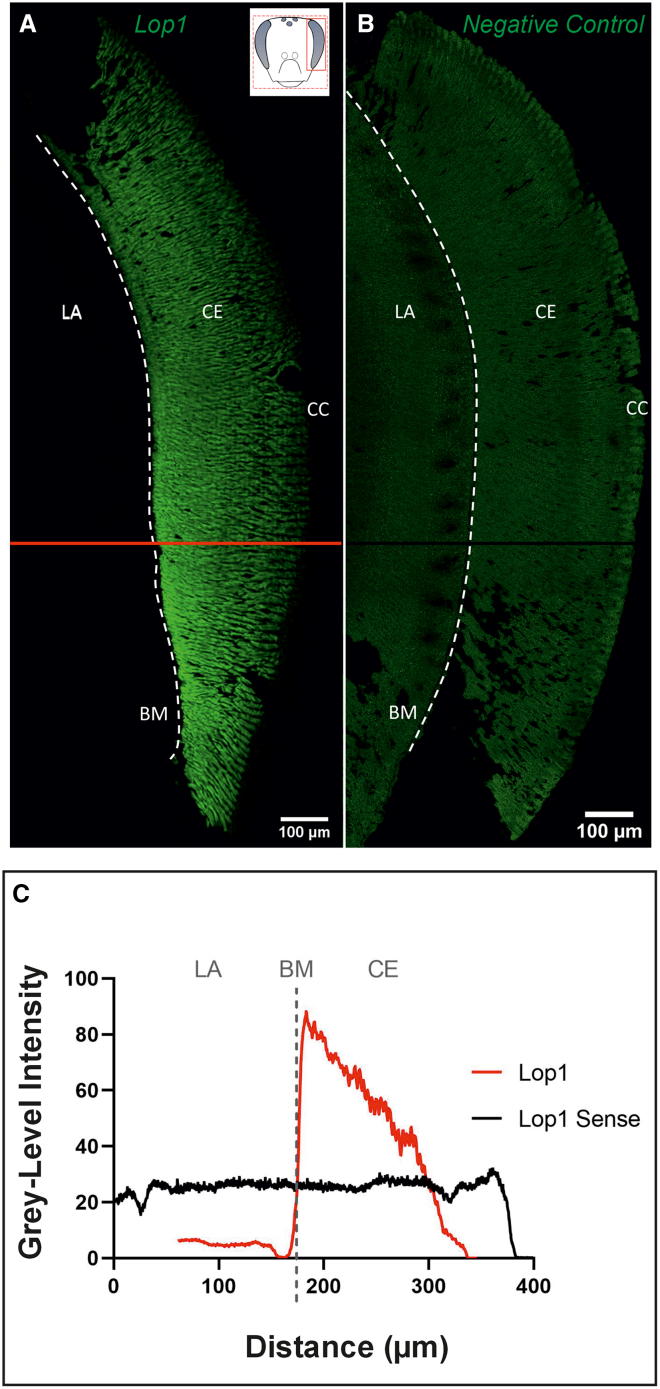
Validation of the fluorescence *in situ* hybridization (FISH) technique used (A) FISH of RNA via the probe encoding the opsin *Lop1;* the light green signal represents the location of the corresponding RNA. (B) FISH of RNA via the *Lop1* sense probe (negative control). (A) and (B) show two frontal sections of a worker compound eye (inset in A). LA: lamina; BM: basement membrane, indicated by a dashed line; CE: compound eye; CC: crystalline cones. Scale: 100 μm. (C) Grey-level intensity (gray value as a function of distance in microns) generated by the *Lop1* probe, quantified at the level of the red line in (A), and by the *Lop1* sense probe, quantified at the level of the black line in (B). Data from one worker.

We first aimed at validating the FISH technique used in our study. We thus compared two worker brains, one labeled with the *Lop1* RNA targeting probe and another labeled with the *Lop1* sense probe as a negative control. The *Lop1* probe yielded an intense signal all along the longitudinal axis of the ommatidia, i.e., between the cells adjacent to the CC and the basement membrane (BM), the demarcation border between the retina and the lamina (LA) (Figure 1A). In contrast, the compound eye labeled with the *Lop1* sense probe generated only autofluorescence (Figure 1B). This lack of signal was also confirmed by the difference in grey-level intensity generated by the two images (Figure 1C), which showed an intensity peak generated by the *Lop1* probe at the BM, which was totally absent in the image labeled with the *Lop1* sense probe. These results confirm the specificity of the *Lop1* signal obtained via the FISH technique.

### Distribution of opsin RNAs in the compound eyes and ocelli of honeybee workers

#### Compound eyes

**Figure 2:**
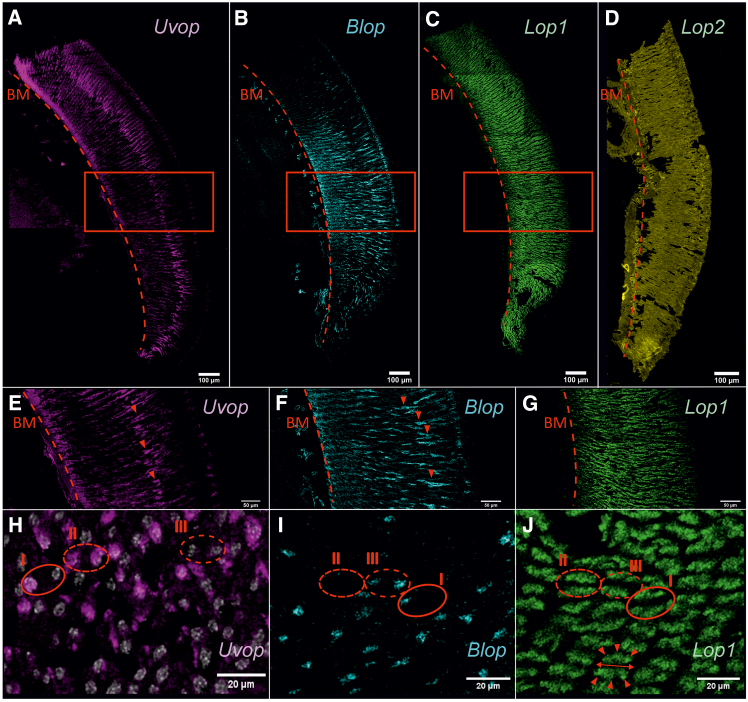
FISH of RNA encoding the opsins *Uvop*, *Blop*, *Lop1*, and *Lop2* in the compound eyes of honeybee workers (A, E, and H) FISH of RNA encoding *Uvop* (magenta signal); (B, F, and I) FISH of RNA encoding *Blop* (cyan signal); (C, G, and J) FISH of RNA encoding *Lop1* (green signal); (D), FISH of RNA encoding *Lop2* (yellow autofluorescence). Sections shown are either frontal (A–G) or sagittal (H–J). The light signal indicates the location of each RNA represented by its respective artificial color. In frontal sections, dashed lines in A–G represent the basement membrane (BM). Red rectangles in A, B, and C corresponds to the zooms shown in E, F, and G, respectively. Red triangles indicate the position of the nuclei of photoreceptors expressing *Uvop* (E) and *Blop* (F). In sagittal sections (H–J), circles delimited by a full red line indicate type I ommatidia, with one photoreceptor labeled with the *Uvop* probe (H) and another with the *Blop* probe (I). Circles delimited by a short-dashed line indicate type II ommatidia, with two photoreceptors labeled with the *Uvop* probe (H) and no label with the *Blop* probe (I). Circles delimited by a long-dashed line indicate type III ommatidia, with two photoreceptors labeled with the *Blop* probe (I) and no label with the *Uvop* probe (H). The *Lop1* probe labels six photoreceptors in each ommatidia (J, red triangles). The double red arrow (J) indicates the assumed position of the *Uvop* or *Blop* labeled photoreceptors. Gray staining corresponds to photoreceptor nuclei labeled with DAPI. The scale is indicated in each panel. Data from five workers (one for A and E; one for B, C, F, and G; one for D; one for H; and one for I and J).

We first examined the general distribution of opsin RNAs within the compound eyes of workers and their specific distribution within single ommatidia. FISH performed on frontal sections of the compound eyes showed a labeling of *Uvop* RNAs around the nuclei of photoreceptor cells as well as extensions on both sides of these nuclei (Figures 2A and 2E). Labeling of *Blop* RNAs showed a similar distribution to that of *Uvop* RNAs, with more prominent extensions along the ommatidia (Figures 2B and 2F). As in the previous staining (Figure 1), we observed a homogeneous distribution of *Lop1* RNAs throughout the compound eye (Figures 2C and 2G). On the contrary, no signal for *Lop2* RNA could be detected (Figure 2D); only autofluorescence was visible upon labeling with the *Lop2* probe. In addition, sagittal sections allowed visualizing the distribution of individual opsin RNAs within single ommatidia and confirmed the presence of the three types of ommatidia previously reported^13^: type I containing one lvf UV-sensitive and one lvf blue-sensitive photoreceptor, type II containing two lvf UV-sensitive photoreceptors and type III containing two lvf blue-sensitive photoreceptors (Figures 2H and 2I). All three types present six svf green-sensitive photoreceptors (Figure 2J). As in a previous study,^13^ the expression pattern of the short ninth photoreceptor cell remained unclear.

**Figure 3:**
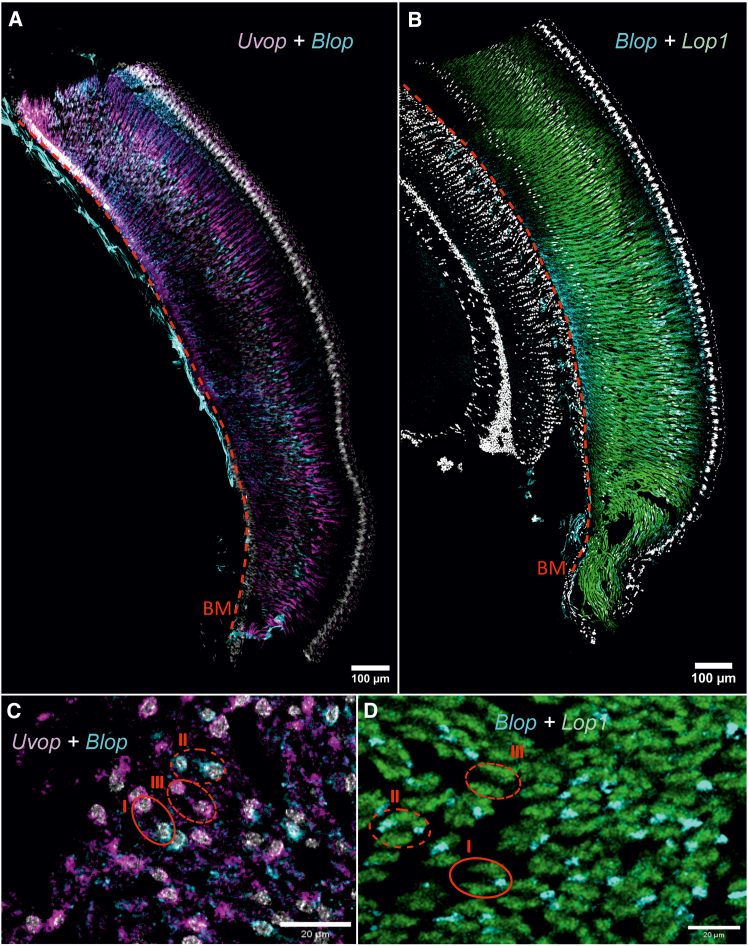
Double labeling of *Uvop* and *Blop* RNA and *Blop* and *Lop1* RNA in the compound eyes of honeybee workers (A and C) Labeling of *Uvop* and *Blop* RNA (magenta and cyan signal, respectively) in frontal (A) and sagittal (C) sections of compound eyes. (B and D) Labeling of *Blop* and *Lop1* RNA (cyan and green signal, respectively) in frontal (B) and sagittal (D) sections of compound eyes. The white-greyish staining corresponds to the labeling of nuclei with DAPI. The red dashed line in A and B represents the basement membrane (BM). Circles delimited by a full red line indicate type I ommatidia, with one photoreceptor labeled with the *Uvop* probe and another with the *Blop* probe (C and D). Circles delimited by a short-dashed line indicate type III ommatidia, with two photoreceptors labeled with the *Uvop* probe and no label with the *Blop* probe (C and D). Circles delimited by a long-dashed line indicate type II ommatidia, with two photoreceptors labeled with the *Blop* probe and no label with the *Uvop* probe (C and D). The *Lop1* probe labels six photoreceptors in each ommatidia (D). The scale is indicated in each panel. Data from two workers (one for A and B and one for C and D).

We then performed double labeling to visualize the co-localization of all RNAs of interest. We were able to confirm the more localized expression of *Uvop* and *Blop* RNAs (Figure 3A), whereas *Lop1* RNAs appeared widely distributed throughout the longitudinal axis of the ommatidia (Figure 3B). The double-labeling showed, in the same picture, the presence of the different target RNAs within single ommatidia and thus confirmed the existence of the three classes of ommatidia already mentioned (Figures 3C and 3D). The proportions of each ommatidial type were estimated across the entire compound eye of worker bees. We identified 44.0% type I, 30.5% type II, and 25.2% type III ommatidia. This distribution corresponds to 52.7% UV-sensitive and 47.3% blue-sensitive photoreceptors (see Table 1).

**Table 1:** Percentages of the three ommatidial types and the corresponding proportions of UV- and blue-sensitive photoreceptors in the compound eyes of the three honeybee castes: workers, queens, and drones

Caste	Ommatidial Types (%)			Photoreceptor Type (%)	
	Type I	Type II	Type III	UV	Blue
**Workers**	44.3	30.5	25.2	52.7	47.3
**Queens**	43.2	31.8	25.0	53.4	46.6
**Drones (VA)**	45.5	36.3	18.2	59.1	40.9
**Drones (DA)**	50.9	49.1	–	18.6	81.4

For drones, data are presented separately for the ventral area (VA) and the dorsal area (DA) of the compound eye.

#### Ocelli

**Figure 4:**
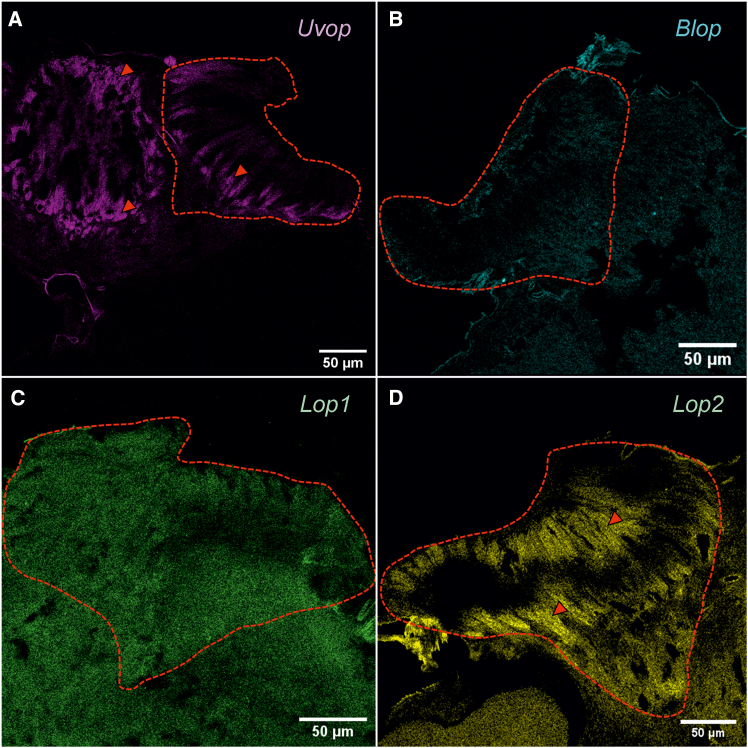
FISH of RNA encoding the opsins *Uvop*, *Blop*, *Lop1*, and *Lop2* in frontal sections of the ocelli of honeybee workers (A) FISH of RNA encoding *Uvop* (magenta signal); (B) FISH of RNA encoding *Blop* (blue autofluorescence); (C) FISH of RNA encoding *Lop1* (green autofluorescence); (D), FISH of RNA encoding *Lop2* (yellow signal). Red dashed lines delimit a lateral ocellus; red triangles indicate the labeling of photoreceptors expressing *Uvop* (A) and *Lop2* (D). Scale bars: 50 μm. Data from four workers (each panel a different individual).

**Figure 5:**
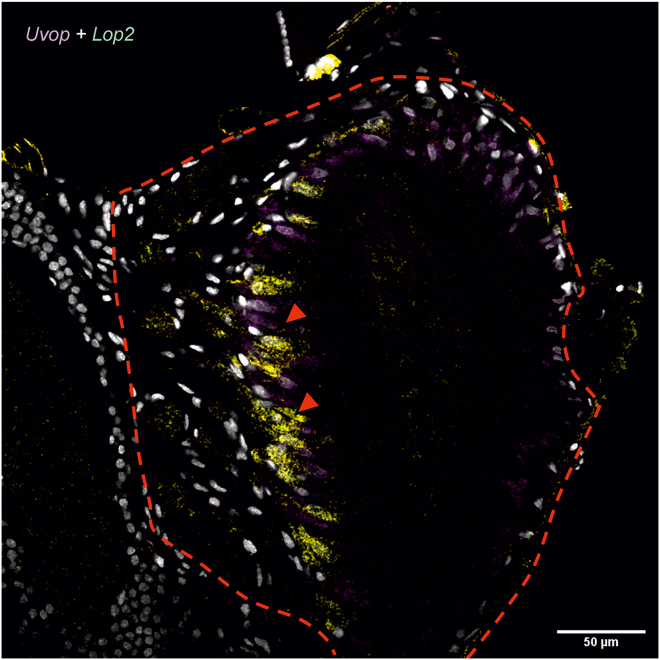
Double labeling of *Uvop* and *Lop2* RNA in the ocelli of honeybee workers Double labeling of *Uvop* (magenta signal) and *Lop2* (yellow signal) RNA in frontal sections of the lateral ocelli in workers. The white-greyish staining corresponds to the labeling of nuclei with DAPI. The red dashed lines delimits a lateral ocellus; red triangles indicate the labeling of photoreceptors expressing *Uvop* and *Lop2*. Data from one worker.

A strong signal could be observed upon labeling with the *Uvop* (Figure 4A) and the *Lop2* probes (Figure 4D), thus revealing the presence of UV and green-sensitive photoreceptors. On the contrary, no signal could be detected for the *Blop* (Figure 4B) and the *Lop1* probes (Figure 4C), except for the autofluorescence generated by the bee brain. The double *Uvop/Lop2* labeling revealed a spatial alternation of photoreceptors expressing the two RNAs (Figure 5).

### Distribution of opsin RNAs in the compound eyes and ocelli of honeybee queens

#### Compound eyes

**Figure 6:**
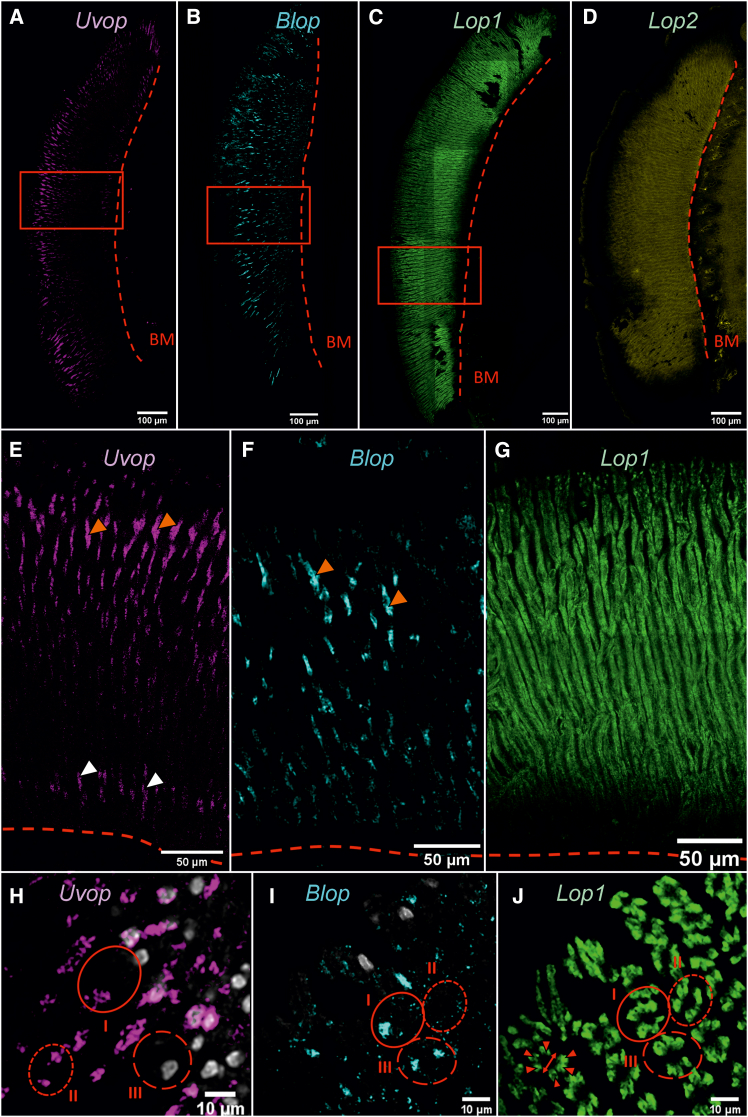
FISH of RNA encoding the opsins *Uvop*, *Blop*, *Lop1*, and *Lop2* in the compound eyes of honeybee queens (A, E, and H) FISH of RNA encoding *Uvop* (magenta signal); (B, F, and I) FISH of RNA encoding *Blop* (cyan signal); (C, G, and J) FISH of RNA encoding *Lop1* (green signal); D), FISH of RNA encoding *Lop2* (yellow autofluorescence). Sections shown are either frontal (A–G) or sagittal (H–J). The light signal indicates the location of each RNA represented by its respective artificial color. In frontal sections, dashed lines in A–G represent the basement membrane (BM). Red rectangles in A, B, and C correspond to the zooms shown in E, F, and G, respectively. Orange triangles indicate the position of the nuclei of photoreceptors expressing *Uvop* (E) and *Blop* (F). In sagittal sections (H–J), circles delimited by a full red line indicate type I ommatidia, with one photoreceptor labeled with the *Uvop* probe (H) and another with the *Blop* probe (I). Circles delimited by a short-dashed line indicate type II ommatidia, with two photoreceptors labeled with the *Uvop* probe (H) and no label with the *Blop* probe (I). Circles delimited by a long-dashed line indicate type III ommatidia, with two photoreceptors labeled with the *Blop* probe (I) and no label with the *Uvop* probe (H). The *Lop1* probe labels six photoreceptors in each ommatidia (J, red triangles). The double red arrow (J) indicates the assumed position of the *Uvop* or *Blop* labeled photoreceptors. Gray staining corresponds to photoreceptor nuclei labeled with DAPI. The scale is indicated in each panel. Data from three queens (one for A, B, E, F, H, and I; one for C,G, and J and one for D).

We first examined the general distribution of opsin RNAs within the compound eyes of queens as well as their specific distribution within single ommatidia. Fertilized queens obtained from our apiary were used throughout. Expression patterns of opsin RNAs strongly resembled those of workers. Single-probe labeling revealed a localized distribution of *Uvop* and *Blop* RNAs close to the nuclei of the two lvf photoreceptors (R1 and R5). A more extended expression was found in the case of *Blop* (Figures 6A, 6B, 6E, and 6F). As in workers, labeling of *Lop1* RNA was localized in the six svf photoreceptors (R2-R4 and R6-R8) and extended over the entire compound eye of queens (Figures 6C and G) and stretched all along the longitudinal axis of ommatidia. On the contrary, *Lop2* labeling yielded no signal and only autofluorescence generated by the brain was detectable in this case (Figure 6D).

A relevant difference with the worker pattern concerned the ninth basal photoreceptor, whose nature remained unclear in previous works^13^ and in our labeling of the worker compound eye and ommatidia. In queens, this short basal photoreceptor was clearly labeled by the *Uvop* probe, at least in the upper part of the compound eye, thus indicating that it is a UV sensitive (Figures 6A and 6E). This *Uvop* signal was also detected in the lower part of the eye but was not strong enough to be clearly discernible.

Sagittal sections showed that the opsin pattern present in single ommatidia corresponded to that of workers. The same three types of ommatidia were found: type I, with one photoreceptor expressing *Uvop* RNA and another expressing *Blop* RNA (Figures 6H and 6I), type II, with two photoreceptors expressing *Uvop* RNA (Figure 6H), and type III with two photoreceptors expressing *Blop* RNA (Figure 6I). In all three types, six photoreceptors expressing *Lop1* RNA were found (Figure 6J).

**Figure 7:**
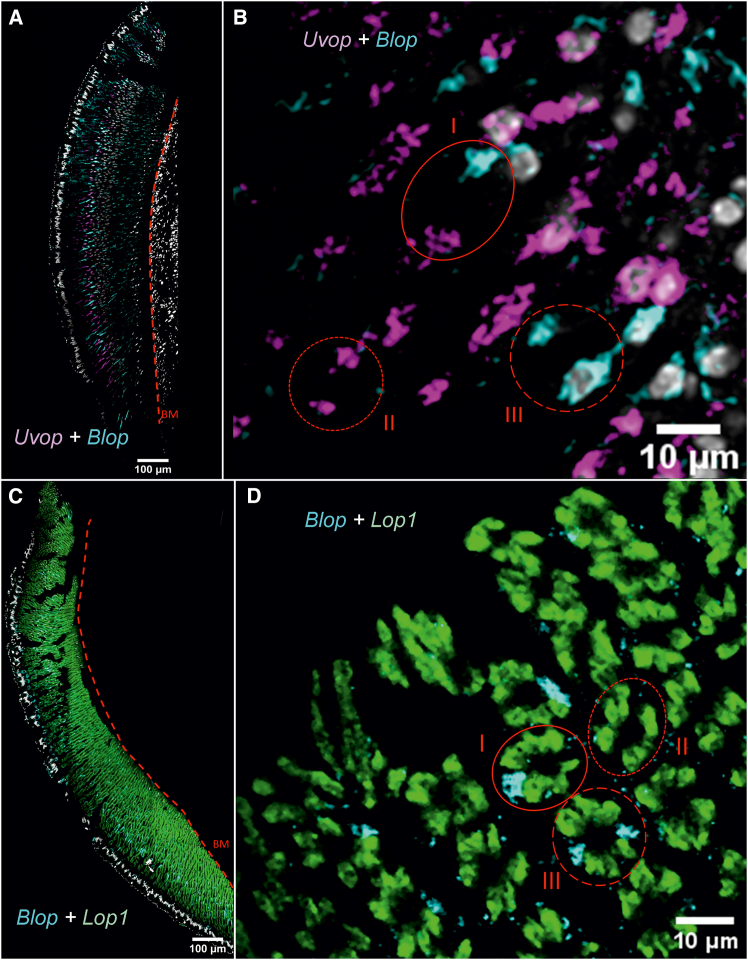
Double labeling of *Uvop* and *Blop* RNA and *Blop* and *Lop1* RNA in the compound eyes of honeybee queens (A and B) Labeling of *Uvop* and *Blop* RNA (magenta and cyan signal, respectively) in frontal (A) and sagittal (B) sections of compound eyes. (C and D) Labeling of *Blop* and *Lop1* RNA (cyan and green signal, respectively) in frontal (C) and sagittal (D) sections of compound eyes. The white-greyish staining corresponds to the labeling of nuclei with DAPI. The red dashed line in A and C represents the basement membrane (BM). Circles delimited by a full red line indicate type I ommatidia, with one photoreceptor labeled with the *Uvop* probe and another with the *Blop* probe (B and D). Circles delimited by a short-dashed line indicate type II ommatidia, with two photoreceptors labeled with the *Uvop* probe and no label with the *Blop* probe (B and D). Circles delimited by a long-dashed line indicate type III ommatidia, with two photoreceptors labeled with the *Blop* probe and no label with the *Uvop* probe (B and D). The *Lop1* probe labels six photoreceptors in each ommatidia (D). The scale is indicated in each panel. Data from two queens (one for A and B and another for C and D).

The double labeling of opsin RNAs in the compound eyes of queens confirmed that *Uvop* and *Blop* RNAs are colocalized with the nuclei of the lvf photoreceptors R1 and R5 (Figures 7A and 7C) while *Lop1* RNA is distributed throughout the compound eye (Figure 7C) and localized in the svf photoreceptors (R2-R4 and R6-R8). Double labeling of single ommatidia demonstrated the presence of all three types of ommatidia described for workers (Figures 7B and 7D), all including six green sensitive photoreceptors (Figure 7D). In this case, the proportions of the three ommatidial types closely matched those observed in workers: 43.2% type I, 31.8% type II, and 25.0% type III ommatidia, confirming a shared pattern among female bees—workers and queens. This similarity was further supported by the photoreceptor distribution, with 53.4% UV-sensitive and 46.6% blue-sensitive photoreceptors (see Table 1).

#### Ocelli

**Figure 8:**
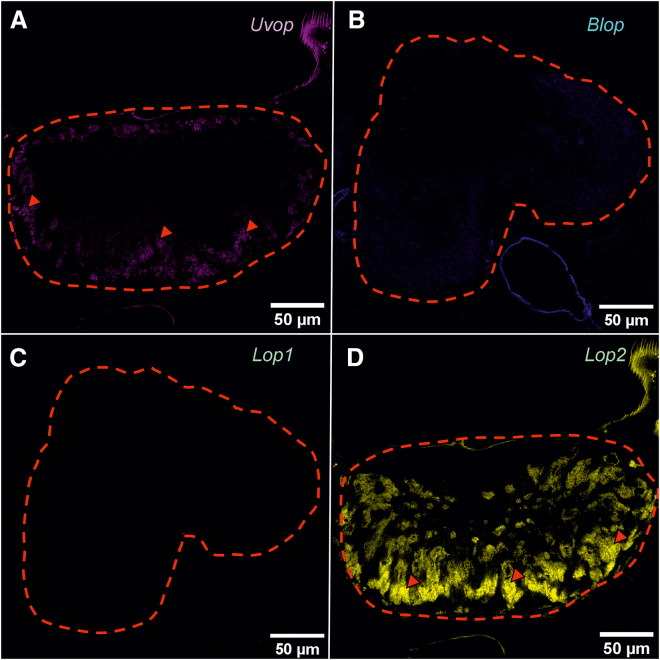
FISH of RNA encoding the opsins *Uvop*, *Blop*, *Lop1*, and *Lop2* in frontal sections of the ocelli of honeybee queens (A) FISH of RNA encoding *Uvop* (magenta signal); (B) FISH of RNA encoding *Blop*; (C) FISH of RNA encoding *Lop1*; (D), FISH of RNA encoding *Lop2* (yellow signal). Red dashed lines delimit a median ocellus; red triangles indicate the labeling of photoreceptors expressing *Uvop* (A) and *Lop2* (D). Scale bars: 50 μm. Data from four queens (one individual per panel).

**Figure 9:**
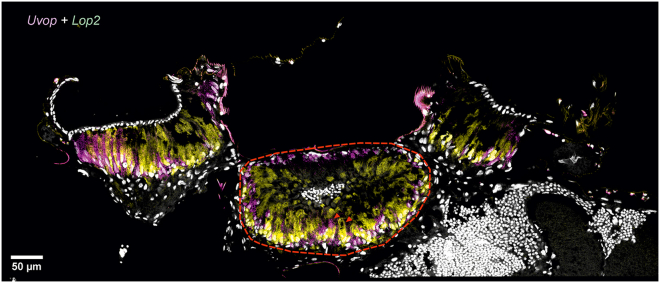
Double labeling of *Uvop* and *Lop2* RNA in the ocelli of honeybee queens Double labeling of *Uvop* (magenta signal) and *Lop2* (yellow signal) RNA in frontal sections of the three ocelli in fertilized queens. The white-greyish staining corresponds to the labeling of nuclei with DAPI. The red dashed line demarcates the median ocelli. The red triangles indicate the labeling of photoreceptors expressing *Uvop* and *Lop2*. Scale bars, 50 μm. Data from one queen.

In the ocelli, a pattern of opsin RNA expression similar to that of workers was found, i.e., photoreceptors expressing *Uvop* (Figure 8A) and *Lop2* (Figure 8D) were detected, but neither *Blop* (Figure 8B) nor *Lop1* RNA expression (Figure 8C) was observed. The double labeling with *Uvop* and *Lop2* probes revealed a mutual exclusion of photoreceptors expressing *Uvop* and *Lop2* (Figure 9).

### Distribution of opsin RNAs in the compound eyes and ocelli of honeybee drones

#### Compound eyes

**Figure 10:**
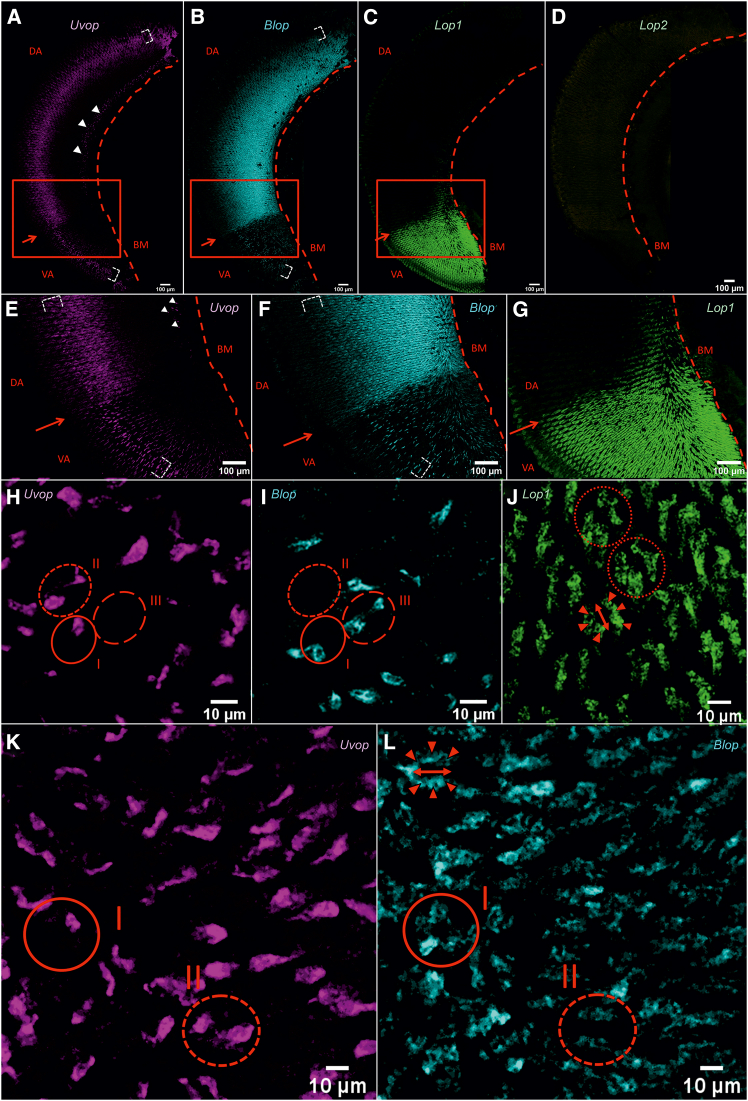
FISH of RNA encoding the opsins *Uvop*, *Blop*, *Lop1*, and *Lop2* in the compound eyes of honeybee drones (A, E, H, and K) FISH of RNA encoding *Uvop* (magenta signal); (B, F, I, and L) FISH of RNA encoding *Blop* (cyan signal); (C, G, and J) FISH of RNA encoding *Lop1* (green signal); (D), FISH of RNA encoding *Lop2* (yellow autofluorescence). Sections shown are either frontal (A–G) or sagittal (H–L). The light signal indicates the location of each RNA represented by its respective artificial color. In frontal sections, dashed lines in A–G represent the basement membrane (BM). Red rectangles in A, B, and C correspond to the zooms shown in E, F, and G, respectively. The white brackets in A, B, E, and F indicate the region hosting the nuclei of the eight photoreceptors, overlaid with *Uvop* (A and E) and *Blop* RNA labeling (B and F). The white triangles indicate the location of the nuclei of the 9^th^ photoreceptor cell expressing *Uvop* (A and E). The red arrows indicate the demarcation between the ventral area (VA) and the dorsal area (DA) of the drone compound eye. Sagittal section in H–J were obtained in the VA while sagittal sections in K and L were obtained in the DA. In the VA, circles delimited by a full red line indicate type I ommatidia, with one photoreceptor labeled with the *Uvop* probe (H) and another with the *Blop* probe (I). Circles delimited by a short-dashed line indicate type II ommatidia, with two photoreceptors labeled with the *Uvop* probe (H) and no label with the *Blop* probe (I). Circles delimited by a long-dashed line indicate type III ommatidia, with two photoreceptors labeled with the *Blop* probe (I) and no label with the *Uvop* probe (H). The *Lop1* probe labels six photoreceptors in each ommatidial type of the VA (J, red triangles). The double red arrow (J) indicates the assumed position of the remaining *Uvop* or *Blop* photoreceptors in the VA. In the DA, circles delimited by a full red line indicate type I ommatidia, with one photoreceptor labeled with the *Uvop* probe (K) and the other with seven photoreceptors labeled with the *Blop* probe (L). Circles delimited by a short-dashed line indicate type II ommatidia, with two photoreceptors labeled with the *Uvop* probe and six photoreceptors labeled with the *Blop* probe (K). Thus, each ommatidia in the drone DA contains six photoreceptors labeled with the *Blop* probe (L, red triangles). The double red arrow (L) indicates the assumed position of the remaining *Uvop* or *Blop* photoreceptors in the DA. Data from three drones (one for A, B, E, F, H, I, K, and L; one for C, G, and J and one for D).

We next focused on opsin RNA distribution in the compound eyes and in single ommatidia of drones. The FISH technique revealed a clear division of the retina into two different areas, a lower ventral one (VA) and a dorsal one (DA) (Figures 10A–10C, 10E–10G). In the VA, RNAs labeled with the *Uvop*, *Blop*, and *Lop1* probes showed an organization similar to that of the compound eyes of female workers and queens, i.e., *Uvop* and *Blop* labeling around the nuclei of the lvf R1 and R5 photoreceptors (Figures 10A, 10B, 10E, and 10F) with larger extensions for *Blop*, projecting to the BM, while *Lop1* labeling was widely distributed throughout the ventral area and all along the longitudinal axis of the svf photoreceptors R2-R4 and R6-R8 located in the VA (Figures 10C–10G).

The picture was significantly different in the DA where a significant and more diffuse presence of *Uvop* RNA was observable compared to the VA (Figures 10A–10E). A clear *Uvop* labeling was also apparent for the ninth photoreceptor adjacent to the basal membrane (Figures 10A–10E, white triangles), thus confirming the UV-sensitive nature of this photoreceptor in the DA of drones. The localization of *Blop* RNA was also very diffuse within the DA (Figures 10B–10F). Remarkably, the DA did not show any labeling of *Lop1* RNA (Figures 10C–10G), thus revealing a total absence of green-sensitive photoreceptors in the dorsal region of the compound eye.

Sagittal sections allowed us to visualize opsin RNA distribution within single ommatidia. In the VA, the labeling confirmed the presence of the three types of ommatidia found in workers and queens, i.e., type I containing one UV-sensitive and one blue-sensitive photoreceptor, type II containing two UV-sensitive photoreceptors, and type III containing two blue-sensitive photoreceptors (Figures 10H and 10I). As in workers and queens, all three ommatidial types contain six svf green-sensitive photoreceptors (Figure 10J). In the DA, the picture was entirely different. Two ommatidial types were found in this region: type I, which contained one photoreceptor expressing *Uvop* and type II, which comprised two photoreceptors expressing *Uvop* (Figure 10K). Importantly, and contrary to VA ommatidia, no photoreceptor expressing *Lop1* was found. In the DA, the six svf photoreceptors that expressed *Lop1* in the VA were replaced by six photoreceptors expressing *Blop* (Figure 10L, red triangles). Thus, type I ommatidia contain one photoreceptor expressing *Uvop* and seven photoreceptors expressing *Blop*, while type II ommatidia contained two photoreceptors expressing *Uvop* and six photoreceptors expressing *Blop*. In the case of the *Lop2* probe, only autofluorescence was detected (Figure 10D).

**Figure 11:**
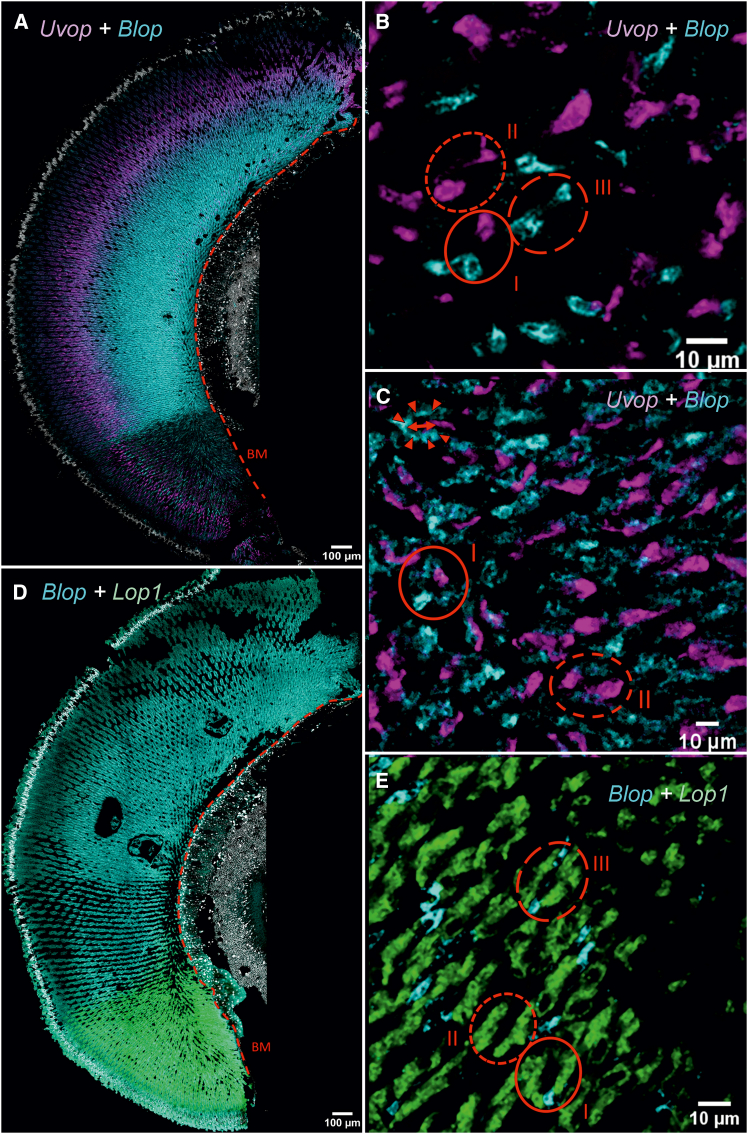
Double labeling of *Uvop* and *Blop* RNA and *Blop* and *Lop1* RNA in the compound eyes of honeybee drones (A, B, and C) Labeling of *Uvop* and *Blop* RNA (magenta and cyan signal, respectively) in frontal (A) and sagittal (B and C) sections of compound eyes. (D and E) Labeling of *Blop* and *Lop1* RNA (cyan and green signal, respectively) in frontal (D) and sagittal (E) sections of compound eyes. The white staining corresponds to the labeling of nuclei with DAPI. The red dashed lines (A and D) represent the basement membrane (BM). Figures (B) and (E) were obtained in the VA. Circles delimited by a full red line indicate type I ommatidia, with one photoreceptor labeled with the *Uvop* probe (B) and another with the *Blop* probe (B and E). Circles delimited by a short-dashed line indicate type II ommatidia, with two photoreceptors labeled with the *Uvop* probe (B) and no label with the *Blop* probe (B and E). Circles delimited by a long-dashed line indicate type III ommatidia, with two photoreceptors labeled with the *Blop* probe (B and E) and no label with the *Uvop* probe (B). The *Lop1* probe labels six photoreceptors in each ommatidial type of the VA (E). Figure (C) was obtained in the DA. Circles delimited by a full red line indicate type I ommatidia, with one photoreceptor labeled with the *Uvop* probe and the other seven with the *Blop* probe (C). Circles delimited by a dashed line indicate type II ommatidia, with two photoreceptors labeled with the *Uvop* probe (C) and the other six with the *Blop* probe. Data from two drones (one for A, B, C, and another for D and E).

Double labeling of *Uvop* and *Blop* expressing RNAs on the one hand and of *Blop* and *Lop1* expressing RNAs on the other hand provided a better view of opsin distribution in the compound eyes of drones (Figure 11). We confirmed the separation of the retina into a VA and a DA, with the VA presenting photoreceptors expressing *Uvop*, *Blop*, and *Lop1* RNAs. The two first were located in the two lvf photoreceptor cells R1 and R5 while *Lop1* labeling was distributed all along the longitudinal ommatidial axis of the six svf photoreceptors R2-R4 and R6-R8 (Figures 11A–11D). In the DA, *Uvop* RNAs were found in the vicinity of the nuclei of lvf photoreceptors, whereas *Blop* RNAs were located between the nuclei of these lvf photoreceptors and the BM. No *Lop1* RNA labeling could be detected in this area (Figures 11A–11D). Sagittal sections of the VA showed a classical opsin RNA distribution within single ommatidia consistent with that existing in workers and queens, i.e., with the presence of the three types of ommatidia, type I comprising one UV-sensitive and one blue-sensitive photoreceptor, type II containing two UV-sensitive photoreceptors and type III containing two blue-sensitive photoreceptors (Figures 11B–11E). In all cases, 6 green-sensitive photoreceptors were observed. Sagittal sections of the DA revealed a different pattern of opsin RNA distribution within single ommatidia with respect to the typical one present in workers, queens and the VA of drones. In this case, no *Lop1* photoreceptor was present so that ommatidial types were restricted to two classes: type 1 expressing one UV sensitive photoreceptor and seven blue sensitive photoreceptors, and type 2 expressing two UV sensitive photoreceptors and six blue sensitive photoreceptors (Figure 11C). In the VA, the proportions of the three ommatidial types were comparable to those observed in workers and queens: 45.5% type I, 36.3% type II, and 18.2% type III ommatidia. This distribution corresponded to 59.1% UV-sensitive and 40.9% blue-sensitive photoreceptors (Table 1). In the DA, the proportions of the two ommatidial types were nearly equivalent with 50.9% of type I and 49.1% of type II. Due to the specific photoreceptor composition of these two types, blue-sensitive photoreceptors were clearly dominant, accounting for 81.4%, while UV-sensitive photoreceptors comprised only 18.6% (Table 1).

#### Ocelli

**Figure 12:**
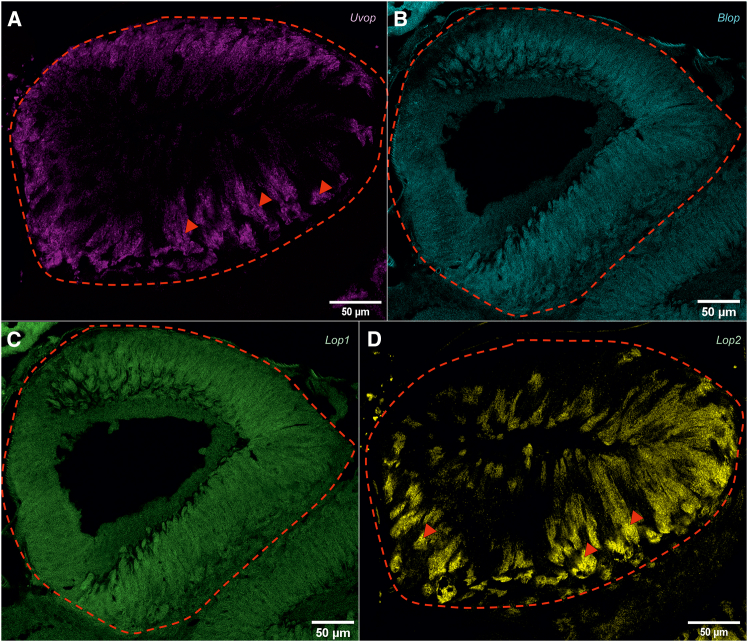
FISH of RNA encoding the opsins *Uvop*, *Blop*, *Lop1*, and *Lop2* in frontal sections of the ocelli of honeybee drones (A) FISH of RNA encoding *Uvop* (magenta signal); (B) FISH of RNA encoding *Blop*; only blue autofluorescence was detected; (C) FISH of RNA encoding *Lop1*; only green autofluorescence was detected; (D), FISH of RNA encoding *Lop2* (yellow signal). Red dashed lines delimit a lateral ocellus; red triangles indicate the labeling of photoreceptors expressing *Uvop* (A) and *Lop2* (D) RNA. Scale bars: 50 μm. Data from two drones (one for A, D, and another for B and C).

**Figure 13:**
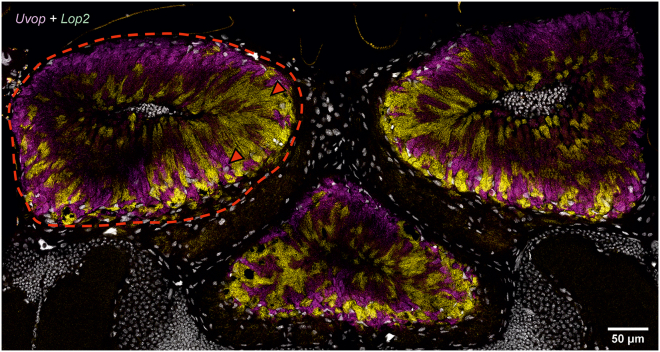
Double labeling of *Uvop* and *Lop2* RNA in the ocelli of honeybee drones Double labeling of *Uvop* (magenta signal) and *Lop2* (yellow signal) RNA in frontal sections of the three ocelli in drones. The white-greyish staining corresponds to the labeling of nuclei with DAPI. The red dashed line demarcates a lateral ocellus. The red triangles indicate the labeling of photoreceptors expressing *Uvop* and *Lop2*. Scale bars, 50 μm. Data from one drone.

Opsin RNA distribution within the drone ocelli was similar to that found in workers and queens, with the presence of photoreceptors expressing *Uvop* and *Lop2* (Figures 12A–12D). As in the other castes, only autofluorescence was detected upon labeling with *Blop* and *Lop1* probes (Figures 12B and 12C). Double labeling of the ocelli confirmed this opsin distribution and revealed a similar pattern to that of workers and queens, i.e., a spatial alternance of *Uvop* and *Lop2* expressing photoreceptors within each ocellus (Figure 13).

## Discussion

**Figure 14:**
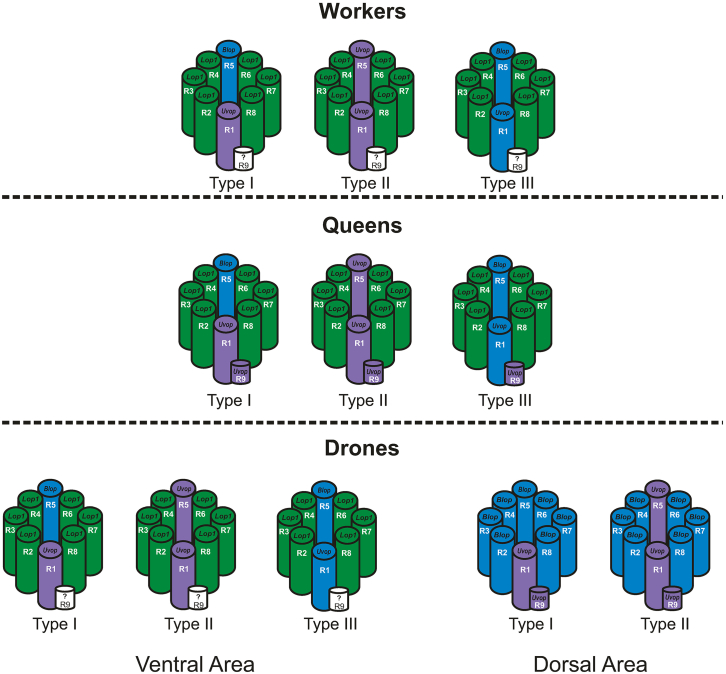
Summary of our findings Ommatidial types are illustrated for the three honeybee castes: workers (top row), queens (middle row), and drones (bottom row). In each case, photoreceptors are labeled R1–R9, and opsin RNA expression (*Uvop*, *Blop*, or *Lop1*) is indicated for each photoreceptor. For drones, the three ommatidial types found in the ventral area are shown on the left, while the two ommatidial types from the dorsal area are shown on the right.

The present study achieved the first simultaneous, comparative mapping of opsin RNAs in the compound eyes and ocelli of the three honeybee castes (workers, drones, and queens) by means of the FISH technique. In addition to the single-probe labeling technique used in previous works,^13^^,^^25^ we implemented a double labeling approach, that strengthened the robustness of our conclusions, particularly with regard to the findings reported here. Our results reveal a similar pattern of opsin distribution in female queens and workers, both in the compound eyes and in the ocelli. In contrast, drones exhibit a markedly different, region-specific distribution of opsins in their compound eyes—most notably in the DA, which encompasses approximately the upper two-thirds of the eye (see Figure 14 for summary).

Previous studies on opsin distribution across ommatidial types in the compound eyes have been performed only in worker bees, specifically by Wakakuwa et al.^13^ Another previous study on opsin expression in worker bees^25^ did not include quantitative analyses of opsin distribution within individual ommatidia; instead, it broadly reported the presence or absence of different opsins in the compound eyes, without referring it to within-ommatidium photoreceptors. This study^25^ did, however, reveal the absence of *Lop2* in the compound eyes of worker bees. Similarly, the work by Lichtenstein et al.,^29^ examined opsin expression in the compound eyes of workers and drones but did not characterize ommatidial types or assess opsin distribution within ommatidial photoreceptors. Moreover, unlike Velarde et al.,^25^ this study did not investigate ocellar opsin expression or report on *Lop2* expression. Therefore, the present study provides a detailed characterization of ommatidial types in terms of opsin expression in queens and drones, with specific attention to the distribution within ommatidial photoreceptors, thereby expanding current knowledge of eye organization in these castes.

### Distribution and organization of opsin RNAs in the compound eyes of the female castes: The case of queens and workers

The study of the distribution of opsin RNAs in the three castes revealed that differences at this level are related to sex and constitute a fascinating example of sexual dimorphism at the level of the insect visual system. This adds to previously reported inter-caste differences in the number of ommatidia,^12^ and is particularly evident in the case of the eight main photoreceptors present in the ommatidia of the DA of drones vs. those of queens and workers, where no strict separation between a VA and a DA was found (the case of the basal ninth photoreceptor will be discussed further). This indicates that the female and male opsin patterns are genetically determined in the female diploid and the male haploid genotypes. The pattern of expression detected in the ocelli was similar in all three castes, with a spatial alternance of ocellar photoreceptors expressing *Uvop* or *Lop2* in the ocellar retina.

The characterization of the opsin distribution in the female compound eye revealed an important presence of *Lop1* RNA in the entire compound eye and a localized distribution of *Uvop* and *Blop* RNAs close to the nuclei of the photoreceptor cells present within ommatidia. The observed difference in the spatial pattern of RNA expression can be explained by the fact that, in both queens and workers, the six photoreceptors expressing *Lop1* RNA correspond to the short visual fiber (svf) photoreceptors that project to the lamina. These cells express higher levels of rhodopsin transcripts, resulting in a broad signal that spans the entire retinal layer. In contrast, the two remaining photoreceptors, which have long visual fibers (lvf) projecting to deeper brain regions, produce lower transcript levels, leading to a more localized signal concentrated around the nuclear layer. Sagittal sections confirmed the presence of the three types of ommatidia described originally by Wakakuwa et al.,^13^ all including six photoreceptors expressing *Lop1* and either one photoreceptor expressing *Uvop* and another expressing *Blop* (type I), two photoreceptors expressing *Uvop* (type II) or two photoreceptors expressing *Blop* (type III).

The dominance of *Lop1* expressing photoreceptors in the compound eyes may be related to the importance of the L-receptor type for motion and distance estimation,^32^ motion parallax,^33^^,^^34^ and edge detection,^35^ which are of crucial importance for a flying insect. Experiments with free-flying bees have shown that differences in retinal speed, which are used to gauge distances to objects, are perceived via L-receptor contrast.^36^^,^^37^ In other words, navigation efficiency relies on modulated signals of L-receptors. It could be, therefore, adaptive to expand the number of *Lop1*-expressing photoreceptors for these navigational purposes. Furthermore, L-receptor contrast perceived via *Lop1-*expressing photoreceptors mediates distant object detection,^38^^,^^39^^,^^40^^,^^41^ which reaffirms the importance of green-sensitive photoreceptors in the case of a flying insect.

The similarity between the patterns of opsin distribution in workers and queens in the peripheral visual system was not necessarily predictable. Differences in opsin distribution could have been expected because queens spend most of their life in the darkness of the hive and restrict their experience of the external world to the short mating flight. On the contrary, worker bees perform hundreds of foraging flights in natural environments. The fact that queens have fewer ommatidia than workers^12^ is consistent with this difference in life styles. Thus, it can be hypothesized that RNA expression patterns do not differ within the same sex, i.e., between queens and worker bees. As our analyses were performed on mated queens, having already completed their nuptial flights, it would be interesting to perform the same analyses on younger non-mated queens to determine if there are differences in opsin expression and distribution between these two types of queens.

### Distribution and organization of opsin RNAs in the compound eyes of the male caste: The case of drones

The pattern of opsin distribution present in drones showed the existence of two areas clearly segregated in the retina of compound eyes, a dorsal one, occupying approximately two-thirds of the compound eye and a ventral one, occupying the remaining third. This segregation was already reported by Velarde et al.,^25^ yet without the details provided in our work. The DA of the drone compound eye (DA) included photoreceptors expressing exclusively *Uvop* and *Blop*, thus revealing a dramatic change with respect to the female pattern of opsin distribution. Indeed, in the drone DA, ommatidial types were restricted to two classes given the absence of *Lop1* expressing photoreceptors: type I, which expresses one UV sensitive photoreceptor and seven blue sensitive photoreceptors, and type II, which expresses two UV sensitive photoreceptors and six blue sensitive photoreceptors. Moreover, *Uvop* RNAs were predominantly located close to the nuclei of the photoreceptors whereas *Blop* RNAs were spread out between the nuclei and the BM of the compound eye. This differential expression pattern can be attributed to the structural and functional differences between photoreceptors with short visual fibers (svf) and those with long visual fibers (lvf), as previously described for workers and queens (see aforementioned). In drones, the six svf photoreceptors that originally expressed *Lop1* RNA in queens and workers appear to have switched to expressing *Blop*. Due to their higher levels of rhodopsin transcript expression, *Blop* signals are distributed broadly across the entire retinal layer. In contrast, the two remaining lvf photoreceptors—expressing either *Blop* or *Uvop*—exhibit lower transcript levels, resulting in signal accumulation primarily around the nuclear layer.

In contrast to the upper two-thirds of the compound eyes, the ventral third was similar to that of females, with a massive and higher expression of *Lop1* opsins compared to *Uvop* and *Blop* opsin expression, and with the presence of the three types of ommatidia described for workers and queens, all including 6 green-sensitive photoreceptors. This type of segregation is indicative of a specialized use of these two areas in different behavioral tasks or of a differential processing of different sensory cues during the same task. Drones exhibit an increased number of ommatidia in their compound eyes (approximately 9900) compared to those of workers and queens (approximately 5300 and 4600 ommatidia, respectively).^12^ The visual field of drones is also expanded compared to workers and queens as the two compound eyes meet at the dorsal border, and the lateral extension of each eye is larger (2.5 mm versus 1 mm). Moreover, the upper third of the drone compound eye builds a large upwards-looking acute zone with larger facet diameters (30–40 μm), smaller inter-ommatidial angles (1–2°), and larger rhabdoms (2–3 μm^2^), than the remaining two-thirds of the eye (20–30 μm; 2–4°; 0.8–2 μm^2^).^30^^,^^31^ These features have been related to the fact that drones rely highly on vision and require higher spatial acuity for queen detection against the sky during mating flights.^42^ Tracking the tiny spot of a flying queen against sky during nuptial flights is indeed a main goal of the relatively short existence of drones. In this context, the large amount of UV and blue-sensitive photoreceptors in the expanded DA may contribute to this task. The VA, with its increased number of L-sensitive receptors, points downwards during flight and can thus serve to evaluate ventral motion parallax for distance estimation during displacements and for efficient landing at the hive entrance.^43^

The DA of the honeybee drone eye is neither the only specialized dorsal retinal region in insects nor the only example of sexually dimorphic retinal structure.^44^ Several insect species exhibit comparable specializations adapted to their ecological and behavioral demands. In the house fly *Musca domestica*, for instance, the dorsal-frontal region of the male eye represents a zone of highest visual acuity and extensive binocular overlap. This area is specialized for tracking females in flight during courtship^45^ and is commonly referred as a “love spot”.^46^ At the cellular level, this specialization involves a transformation of the R7 photoreceptor into a short visual fiber (svf) type,^47^ a structural modification distinct from that of honeybee drones, in which the specialization likely involves changes in opsin expression rather than photoreceptor morphology. In *Lycaena rubidus* butterflies, sexually dimorphic eyes result from differential regulation of opsin gene expression, aligning with the differing ecological roles of males and females.^48^ Males possess a dorsal eye region composed of ommatidia that express only UV- and blue-sensitive visual pigments, which are specialized for color discrimination. These highly territorial males presumably use this dorsal visual field for dichromatic color vision and to detect flickering airborne rivals.^49^ In contrast, dragonflies of the genus *Sympetrum* exhibit no sexual dimorphism at the level of eye structure. Nonetheless, their compound eyes are functionally divided into dorsal and ventral regions with distinct roles. The dorsal eye region, used for tracking prey against the sky, features ommatidia with yellow screening pigments and a high density of blue-sensitive receptors, providing both high sensitivity and contrast detection.^50^ This region also includes a fovea with small interommatidial angles, enabling precise visual fixation.^51^^,^^52^ In the ventral retina, a high frequency of green receptors likely supports detection of water surfaces.

This non-exhaustive list of examples illustrates that specialized dorsal regions and, in some cases, sexually dimorphic retinal structures are widespread among insects, serving a range of ecologically relevant visual functions. The honeybee drone DA thus fits into a broader pattern of dorsal visual specializations across insect taxa.

### Opsin expression in the 9th photoreceptor cell in the three honeybee castes

The opsin expression of the short 9^th^ photoreceptor located adjacent to the BM of the compound eyes remained elusive in previous labeling attempts performed both in workers and drones.^13^^,^^25^ It was previously assumed that these photoreceptors would be sensitive to UV light^53^ but previous *in situ* hybridization analyses did not confirm this hypothesis.^13^

In worker bees, our labeling experiments could not identify conclusively the opsin expressed in the 9^th^ photoreceptor type. Labeling with *Uvop* and *Blop* probes did not yield clear signals at the level of these photoreceptors, whereas the *Lop1* probe yielded a more salient signal. Yet, this signal was diffuse and not specific to the area of the 9^th^ photoreceptors as it was also present along the entire compound eye. This uncertainty did not apply to the case of queens where a clear *Uvop* labeling was visible in the 9^th^ photoreceptors present in the upper part of the compound eye. The signal from the 9^th^ photoreceptors in the lower part of the queen compound eye was not strong enough to be conclusive, as in the case of workers. Based on these findings, and considering the similarities in opsin distribution between queens and workers, we suggest that the 9^th^ photoreceptor in workers might also express a UV sensitive opsin, confirming the original conclusions of Menzel and Snyder.^53^ However, this suggestion should be treated with caution and awaits future experimental confirmation.

In the DA of drones, where no *Lop1* is detected, the 9^th^ photoreceptor was clearly labeled by the *Uvop* probe, indicating that this photoreceptor is UV sensitive. In the VA of drones, this conclusion could not be sustained. The VA is essentially identical in opsin composition to that of the worker retina, where the identification of the opsin type of the 9^th^ photoreceptor was difficult. The reasons for this difficulty remain unclear and seem to be biological rather than technical given the success attained in queens and drones. Further experiments controlling the age of workers would be necessary to see if certain markings appear clearer during certain developmental stages.^29^

### Opsin distribution in the ocelli of the three honeybee castes: The case of the Lop2 opsin

Honeybees possess three ocelli located on top of their head. Lateral ocelli contain approximately 1100 photoreceptors each, while the median ocellus contains approximately 1350 photoreceptors.^28^ Our results showed that the pattern of opsin expression in these photoreceptors is similar in the three castes of honeybees. In all cases, photoreceptors expressed *Uvop* and *Lop2* and a spatial alternance of these photoreceptor types was observed in the ocellar retina. Our results confirmed the finding reported by Velarde et al.*,*^25^ namely that *Lop2*-expressing photoreceptors are confined to the ocelli of workers. Our analysis revealed that this pattern also applies to queens and drones, for which such data were previously lacking. The functional significance of the spatial segregation between *Lop1* and *Lop2* remains unclear.

Interestingly, the presence of a second *Lop* opsin, besides the one known for the worker compound eyes, was first detected by phylogenetic analysis of Hymenoptera opsin sequences that aimed at identifying an early gene duplication event within the insect long-wavelength sensitive opsin family.^54^ This study focused on five hymenopteran species (*Bombus impatiens*, *B. terrestris*, *Diadasia afflicta*, *D. rinconis*, and *Osmia rufa*), which did not include *Apis mellifera*, and isolated two *Lop* opsin gene sequences from each of the five species considered. It was suggested that *Lop2* evolves at a slower rate than *Lop1* and, therefore, may be a useful marker for higher-level hymenopteran systematics. This study could not determine where *Lop2* was expressed, so that it was speculated that this opsin may have a specialized function as an extraretinal opsin, expressed in a small number of light-sensitive neurons playing a role in circadian rhythms in the brains of bees.^54^ Two year later, Velarde et al.^25^ reported that *Lop2* was expressed in the ocelli.

Electrophysiological recordings coupled with stimulation with a series of monochromatic flashes (340–600 nm)^28^ allowed characterizing the spectral sensitivity of ocellar photoreceptors. These measurements showed two classes of ocellar photoreceptors in workers, consistent with the pattern of opsin expression reported in our work. The spectral sensitivity of one class peaks at 360 nm (UV sensitive),^28^ which is close to the sensitivity peak exhibited by ommatidial UV photoreceptors (344 nm, Δ_λ_ = 16 nm),^55^ while that of the other class peaks at 500 nm (green sensitive),^28^ which represents an important shift toward shorter wavelengths when compared to the sensitivity of ommatidial green photoreceptors (544 nm, Δ_λ_ = 44 nm).^55^

One explanation for this difference in spectral sensitivity may be related to the presence of the blue photoreceptor type in ommatidia, whose sensitivity peaks at 436 nm, i.e., between the maxima exhibited by UV and green photoreceptors.^55^ Displacing the sensitivity of the green ommatidial photoreceptor toward longer wavelengths may have given the opportunity to incorporate a “newer” photoreceptor without an excessive overlap between spectral sensitivity curves. This displacement is particularly important for color vision mediated by compound eyes, because it provides the basis for trichromatic color vision, and thus for a richer color experience of the environment, but also because it allows a higher wavelength discrimination. Indeed, the determination of the Δ_λ_ function in honeybees shows that wavelengths at which bees achieve the best wavelength discrimination are located at the intersection between adjacent spectral sensitivity curves.^56^ It has also been shown that floral spectra have steeper curves precisely at these intersections, thus facilitating flower color discrimination.^57^ Thus, shifting green receptor sensitivity toward longer wavelengths in compound-eye ommatidia allows adding another receptor type and expanding the range of chromatic differentiation through a better separation of spectral sensitivity curves and consequently their intersection regions.

This hypothesis needs to be taken cautiously because the electrophysiological analysis of the spectral sensitivity of ocellar photoreceptors requires further refined analyses. Indeed, the measurements performed so far are less precise than those achieved in the case of ommatidial photoreceptors. In the latter case, and following the method established by Menzel and Blakers,^18^ measurements were performed from 300 to 700 nm using 4 nm steps so that the precision of the spectral-sensitivity curves was very high.^55^ For ocellar photoreceptors, measurements were limited to the range between 340 and 600 nm and 20 nm steps were used.^28^ Although these ocellar recordings are extremely valuable as they provide a first electrophysiological characterization of the spectral sensitivity of ocellar photoreceptors, important regions of the bee visual spectrum were absent (i.e., 300–339 nm and 600–650 nm), and the precision of recordings was 5 times lower than that of ommatidial photoreceptors. It would be, therefore, interesting to repeat the characterization of spectral sensitivity in the case of ocellar photoreceptors using a methodology equivalent to that used for ommatidial photoreceptors.

Finally, a relevant question concerns the expression of two opsins, *Lop2* and *Uvop*, in the bee ocelli. A modeling study^58^ proposed a possible explanation for this expression pattern, by stating that a key function of the ocelli is to detect changes in the ratio of short-to long-wavelength radiation under typical daylight conditions, thereby supporting color constancy—the ability to perceive object colors consistently despite changes in illumination—a capacity demonstrated in honeybees.^59^^,^^60^ According to the model, input from the ocelli may contribute to color constancy by providing instantaneous measurements of ambient light characteristics. The study showed that the spectral properties of the ambient light could be reconstructed from the responses of the two photoreceptor classes in the dorsal ocelli, thus contributing to color constancy.

### A comparative perspective on ommatidial types and retinal mosaics across species

Our findings enable a comparative analysis of insect visual systems by relating them to extensive studies on similar features in the fruit fly *Drosophila melanogaster*^44^^,^^61^ and butterflies,^62^ among other insects. Ommatidial subtypes have also been reported in these groups^61^^,^^62^^,^^63^ as well as in other insects^44^^,^^64^^,^^65^^,^^66^; however, they are most comprehensively characterized in fruit flies, honeybees, and certain butterfly species.

In bees, eight photoreceptors (R1–R8) form a fused rhabdom spanning the full thickness of the retina, with an additional small ninth photoreceptor (R9) located proximally, i.e., close to the BM. Six of these photoreceptors (R2–R4 and R6–R8) possess short visual fibers (svf) that project to the lamina. These correspond to the six ***Lop1***-expressing photoreceptors in workers and queens, as well as in the ventral region of the compound eye in drones. In the latter, the svf photoreceptors in the upper two-thirds of the compound eye express ***Blop***. Photoreceptors R1 and R5 have long visual fibers (lvf) and project to the medulla; these two photoreceptors show variable expression patterns (*Uvop* or *Blop*) depending on ommatidial type in workers, queens, and drones (see Table 2). In addition, the proximal R9 short photoreceptors have also long visual fibers.

In *Drosophila*, six photoreceptors (R1–R6) also span the entire retinal thickness, forming an open rhabdom, while two inner photoreceptors (R7 and R8) are centrally located, with R7 positioned distally and R8 proximally. Like in bees, R1–R6 are svf photoreceptors projecting to the lamina, while R7 and R8 are lvf photoreceptors projecting to the medulla. The svf photoreceptors express the broadband rhodopsin **Rh1**, which is sensitive to blue/green light and often associates with a UV-absorbing pigment. As in bees, rhodopsin expression in the two lvf photoreceptors (R7 and R8) varies among ommatidial types,^67^^,^^68^ thus giving origin to two main ommatidial subtypes, which were originally identified based on pale or yellow fluorescence when illuminated with blue light.^69^^,^^70^ In so-called pale ommatidia, R7 expresses the UV-sensitive **Rh3** rhodopsin and R8 expresses the blue-sensitive **Rh5** rhodopsin. In the alternative yellow ommatidia, R7 expresses the UV-sensitive **Rh4** rhodopsin, while R8 expresses a green-sensitive rhodopsin.^71^ These findings led to the suggestion that the two distal honeybee photoreceptors (R1 and R5) are *Drosophila* R7-like photoreceptors while the short R9 bee photoreceptor is a *Drosophila* R8 homolog.^72^^,^^73^ While the similarities between the bee R1 and R5 photoreceptors and the fruit fly R7 photoreceptors are consistent with a degree of functional or evolutionary homology, the correspondence between the bee R9 photoreceptor and the fruit fly R8 photoreceptors should be interpreted with caution. This is primarily due to differences in opsin expression observed in our study. In bees, R9 expresses a UV-sensitive opsin in queens and in the ventral region of drones, which contrasts with the blue- or green-sensitive opsins typically expressed by R8 photoreceptors in *Drosophila*.^71^

Among butterflies, *Papilio xuthus* is one of the most extensively studied species in terms of photoreceptor classes and opsin expression.^62^^,^^74^^,^^75^ Five types of rhodopsins have been identified in this species: one UV-sensitive, one blue-sensitive, and three long-wavelength-sensitive opsins.^74^^,^^76^ Striking homologies with honeybees are evident in the two lvf photoreceptors, R1 and R2, which express either UV- or blue-sensitive rhodopsins and give rise to three ommatidial types: type I, in which one receptor expresses a UV-sensitive rhodopsin and the other a blue-sensitive rhodopsin; type II, with both receptors expressing blue-sensitive rhodopsin; and type III, with both expressing UV-sensitive rhodopsin. However, differences emerge in comparison to the uniform rhodopsin expression observed in the six svf photoreceptors of bees and flies. In *Papilio*, these six photoreceptors express two types of long-wavelength rhodopsins.^62^ Specifically, two of the six photoreceptors express the same long-wavelength rhodopsin as the proximal ninth photoreceptor (R9), while the other four show varied combinations of long-wavelength-sensitive rhodopsins.^62^^,^^76^ The R9 photoreceptor in *Papilio* thus differs from that present in honeybee queens and in the DA of drones, where R9 expresses the UV-sensitive opsin *Uvop*.

The percentages of ommatidial types observed in our study differ somewhat from those previously reported for worker bees.^13^ In that earlier study, the proportions of type I, II, and III ommatidia were approximately 44%, 46%, and 10%, respectively.^13^ In contrast, our findings show 44.3%, 30.5%, and 25.2% for type I, II, and III ommatidia, respectively (see Table 1). Notably, these values are consistent with those observed in queens, supporting the existence of a common pattern of ommatidial distribution among female bees and reinforcing the robustness of our findings. The discrepancy with the previous report may be attributed to differences in the labeling techniques used: Wakakuwa et al.^13^ based their estimations on single labeling, whereas our study employed double labeling, which allows for improved resolution and more accurate identification and quantification of ommatidial types. Previous estimates of the overall proportions of UV- and blue-sensitive photoreceptors in the compound eyes of worker bees were 68% and 32%, respectively.^13^ These values were compared to the distribution of yellow and pale R7 photoreceptors in *Drosophila* (65% and 35%, respectively), leading to the hypothesis that differences in retinal mosaics across species may arise from modifications to a shared molecular program governed by evolutionarily conserved factors.^44^ While this hypothesis is compelling, our new estimates—52.7% UV- and 47.3% blue-sensitive photoreceptors (see Table 1; similar values were observed in queens)—call for caution in interpreting this evolutionary parallel.

### Conclusion

Our study provides the first comparative mapping of opsin types in the peripheral visual system (ocelli and compound eyes) of the three known castes (workers, drones, and queens) of the honeybee *Apis mellifera*. Our results showed that the nature and distribution of opsin RNAs in the ocelli is conserved between the three castes, with *Uvop* and *Lop2* photoreceptors alternating along the ocellar retina. Differences related to sex were found in the case of the distribution of opsin RNA in the compound eye. Females, i.e., queens and workers, shared a common opsin-distribution pattern, which included three types of ommatidia, all with six *Lop1*-expressing photoreceptors, and either one *Uvop* and one *Blop*-expressing photoreceptor (type I), two *Uvop*-expressing photoreceptors (type II) or two *Blop*-expressing photoreceptors (type III). In queens, the 9^th^ short photoreceptor could be identified for the first time as a UV-sensitive photoreceptor, while in workers the sensitivity of this photoreceptor remained unclear. Males, i.e., drones, exhibited a clear regionalization of the compound-eye retina, with a ventral area displaying an ommatidial organization similar to that of workers and queens, and a totally different DA occupying two-thirds of the compound eye, which included only two types of ommatidia: type I with one *Uvop* and seven *Blop*-expressing photoreceptors, and type II with two *Uvop*-expressing photoreceptors and six *Blop-*expressing photoreceptors. The short 9^th^ photoreceptors in the DA of drones could be identified as a UV-sensitive photoreceptor. Overall, these findings raise new questions about the functional value of the opsin patterns detected in the three castes, which will give origin to future experiments addressing some of the hypotheses raised in the Discussion of this work.

### Limitations of the study

Our study did not investigate the pattern of opsin expression or the distribution of ommatidial types in the DRA—a small, specialized region of the compound eye that forms a narrow ribbon along the dorsal margin. DRA ommatidia are oriented toward the sky and are specialized for detecting polarized light, playing a key role in navigation.^24^^,^^77^^,^^78^^,^^79^^,^^80^^,^^81^ Future studies should characterize this region in all three castes to identify both commonalities and differences.

As noted throughout this study, and as previously reported,^13^ the opsin expression pattern of the short ninth (R9) photoreceptor in workers remained unclear. A similar uncertainty exists for the R9 photoreceptor in the ventral region of drone eyes. Further research is needed to determine the opsin expressed in these cases.

From a technical perspective, we initially aimed to employ triple labeling to visualize all three opsin RNAs simultaneously. However, the Cal Fluor Red 610 fluorophore, coupled to the Lop1 probe, could not be reliably detected. Although optimal excitation at 590 nm was tested on the confocal microscope, it primarily revealed pigments, complicating signal interpretation. Moreover, no suitable two-photon excitation wavelength was identified. Among the eight fluorophores offered by the Stellaris Company, we tested the three recommended for multi labeling—Quasar 670, Quasar 570, and Cal Fluor Red 610—in order of descending effectiveness. To successfully achieve triple labeling, alternative techniques such as RNAscope, which employs indirect hybridization and offers a broader selection of fluorophores, may be required.

## Resource availability

### Lead contact

Further inquiries and requests for resources or reagents should be directed to the lead contacts, Isabelle Lafon (isabelle.lafon@cnrs.fr) and Martin Giurfa (martin.giurfa@sorbonne-universite.fr).

### Materials availability

This study did not generate new unique reagents.

### Data and code availability

Data: Data reported in this paper will be shared by the lead contact upon request.

Code: This paper does not report original code.

Other items: Any additional information required to reanalyze the data reported in this paper are available from the lead contact upon request.

## Acknowledgments

We thank K. Arikawa (Sokendai University) and three anonymous reviewers for constructive feedback on the manuscript and M. Paoli, B. Paffhausen, L. Baciadonna, G. Lafon, H. Geng, C. Macri, Y. Lai, and G. de Brito Sanchez for engaging and stimulating discussions. We also thank the European Research Council (ERC) for supporting this project (ERC advanced grant COGNIBRAINS to M.G.), the 10.13039/501100004794CNRS and Sorbonne university for funding.

## Author contributions

A.D. performed the experiments; S.L. performed preliminary experiments, which enabled the research; D.C. and B.R. assisted the staining and imaging analyses; results and conclusions were discussed by all authors. The manuscript was written by A.D., I.L., and M.G. All experiments were supervised by I.L. and M.G. Funding was obtained by M.G. All authors reviewed and approved the final version of the manuscript.

## Declaration of interests

The authors declare no competing interests.

## STAR★Methods

### Key resources table

**Table:** 

REAGENT or RESOURCE	SOURCE	IDENTIFIER
**Antibodies**		

Uvop Quasar 670	NCBI LGC Biosearch Technologies	GenBank: NM_001011605, 1448 pb Cat# No. FC-1065
Blop Quasar 570	NCBI LGC Biosearch Technologies	GenBank: NM_001011606, 1757 pb Cat# No. FC-1063
Lop1 Quasar 670	NCBI LGC Biosearch Technologies	GenBank: NM_001011639, 1806 pb Cat# No. FC-1065
Lop2 Quasar 570	NCBI LGC Biosearch Technologies	GenBank: NM_001077825, 1158 pb Cat# No. FC-1063

**Biological samples**		

Compound eyes and ocelli from honeybee (*Apis mellifera*) workers, queens and drones	University apiary located at the University Paul Sabatier, Toulouse.	

**Chemicals, peptides, and recombinant proteins**		

Stellaris RNA FISH Hybridization Buffer	LGC Biosearch Technologies	Cat# SMF-HB1-10
Stellaris RNA FISH Wash Buffer A	LGC Biosearch Technologies	Cat# SMF-WA1-60
Stellaris RNA FISH Wash Buffer B	LGC Biosearch Technologies	Cat# SMF-WB1-20
VECTASHIELD Antifade Mounting Medium	Vector Laboratories	Cat. No. H-1000
Paraformaldehyde	Thermo Fisher Scientific	Cat# AAJ19943K2
Tissue-Tek® OCT Compound	Sakura Finetek	Cat# 4583
DAPI (4′,6-diamidino-2-phenylindole)	Thermo Fisher Scientific	Cat# D1306

**Experimental models: Organisms/strains**		

Honeybee (*Apis mellifera*) workers, queens and drones	University apiary located at the University Paul Sabatier, Toulouse	

**Software and algorithms**		

Stellaris Probe Designer	Biosearch Technologies	Version 4.2
Corel Draw2024	Corel Corporation	Version 25.2.1.313
FIJI (ImageJ)	Schindelin et al., 2012	https://fiji.sc
GraphPad Prism 8	GraphPad Software	RRID:SCR_002798

### Experimental model and study participant details

Experiments were carried out on adult workers, drones and fertilized queens of European honeybees *Apis mellifera*. All bees were collected from colonies located in the apiary of the Research Center on Animal Cognition (Toulouse, France). Worker bees were collected at a feeder placed in the apiary and enclosed in glass tubes before anesthetizing them on ice for 10 min; drones and queens were collected by hand. Data obtained from 13 workers, 10 queens and 8 drones are shown.

### Method details

#### Sequence analysis of opsins RNA

The gene sequences used to synthesize the probes of the different opsins were downloaded from the National Center for Biotechnology Information (NCBI) where the identifiers of *Uvop* (NM_001011605, 1448 pb), *Blop* (NM_001011606, 1757 pb), *Lop1* (NM_001011639, 1806 pb) and *Lop2* (NM_001077825, 1158 pb) are available.

#### Probe set design

The software “Stellaris Probe Designer” of Biosearch Technologies was used to synthesize the complementary probes to the different RNA (https://www.biosearchtech.com/stellaris-designer). This software allows, from an input sequence (FASTA format for example), to synthesize about fifty very specific oligonucleotides distributed all along the target sequence, each containing about 20 nucleotides, in order to obtain an optimal specific binding. Moreover, each of the fifty oligos is coupled to the same fluorophore allowing the visualization of the targeted RNA. Finally, a list of all the synthesized oligos was generated as well as a graph representing the position of each oligo on the target sequence. Our aim was to observe all opsins via triple labeling and therefore we initially used all the antisense probes for the sequences of *Uvop* (Quasar 670), *Blop* (Quasar 570), *Lop1* (CAL Fluor Red 610) and *Lop2* (Quasar 570). However, imaging difficulties prevented us from visualizing the Cal Fluor Red 610 fluorophore in the double-labelling experiments. To address this problem, we used the antisense probes for *Lop1* and *Uvop* in Quasar 670 and the antisense probes for *Blop* and *Lop2* in Quasar 570. We also used a sense probe for the *Lop1* sequence (Quasar 670) as a negative control. Under these conditions, our study allowed to visualize two opsin types at the same time in the same preparation.

#### Dissection and tissue preparation

After anesthesia, bee heads were opened, separated from the body and kept in 4% paraformaldehyde (PFA) solution during one night. After the incubation, brains were dissected under binocular microscope and placed in 4% PFA for at least 2 to 4 h. Once the tissue fixation ended, dissected brains were included in 15% sucrose solution for at least half a day (maximum one full day), then placed in 30% sucrose solution overnight. Brains were then frozen at −80°C after OCT inclusion and cut in the cryostat (Leica CM3050 S) on the following day. Frontal and sagittal sections, 30 μm thick, were realized at −21°C.

#### Fluorescence *in situ* hybridization of RNA

A protocol from Stellaris, Biosearch Technologies, was used. The first step consisted in the incubation of slices in 400 μL of a fixation buffer (37% formaldehyde, PBS RNase-free and Nuclease-free water). After two washes in PBS, slices were dehydrated in 70% ethanol for 1 h at room temperature. After the dehydration, slices were placed in a humidified chamber (a box containing a paper with clean water), washed with 200 μL of buffer A (10 mL - for 100 mL - of Stellaris RNA FISH 5X Wash Buffer A, 5 mL – for 50 mL – of deionized formamide and 35 mL – for 350 mL – of nuclease-free water, for a total volume of 50 mL) and placed at 37°C during 30 min. Then, buffer A was removed and slices were incubated from 4 to 16 h with 200 μL of hybridization solution (“Stellaris RNA FISH Hybridization Buffer”, formamide and 2μL of the opsin probe targeted). A clean cover glass was positioned on each slide allowing the distribution of the hybridization solution on the tissue.

Next, the slides were washed and incubated with 200 μL of buffer A for 30 min at 37°C and DAPI solution (5μg/mL + PBS), used to counterstain nuclei, also with an incubation of 200 μL per slide for 30 min at 37°C. Slides were then washed with 200 μL of buffer B (88 mL of nuclease-free water) and mounted with a drop of Vectashield covered with a clean cover glass sealed with clear nail polish.

#### Single labeling

Single labelling was performed to visualize the specific expression of each type of opsin. This labeling served as an initial qualitative control to validate the specificity of antisense probes designed using the Stellaris Probe Designer tool (Biosearch Technologies), based on sequences retrieved from the NCBI database (*Uvop, Blop, Lop1*, and *Lop2*).

Hybridization was carried out on 30 μm-thick brain sections, prepared as described above. After incubation in Buffer A, the tissues were exposed to a hybridization solution containing the antisense probes labeled with the corresponding fluorophores: Quasar 670 for *Uvop* and *Lop1*, and Quasar 570 for *Blop* and *Lop2*. The slides were then washed, counterstained with DAPI (5 μg/mL), and mounted with Vectashield.

This step allowed for the assessment of the localization and signal intensity of each opsin individually, and for the optimization of imaging parameters (laser power, detector gain) on the Leica SP8 confocal microscope. The resulting images served as a reference for subsequent double labeling experiments. A negative control was performed by hybridizing a sense probe for *Lop1*, which showed no specific signal, confirming the absence of nonspecific probe binding.

#### Double labeling

Double labelling was used to simultaneously visualize two types of opsin RNAs within the same tissue section and examine their potential co-expression and spatial distribution. Due to imaging limitations with the fluorophore CAL Fluor Red 610 (initially used for *Lop1*), a new fluorophore distribution strategy was applied. Antisense probes for *Lop1* and *Uvop* were labeled with Quasar 670 (excitation at 644 nm), while probes for *Blop* and *Lop2* were labeled with Quasar 570 (excitation at 555 nm). This configuration enabled simultaneous visualization of *Uvop + Blop* or *Blop + Lop1* in compound eye sections, and of *Uvop* and *Lop2* in ocellar sections.

Hybridization was performed on 30 μm-thick brain sections, prepared as described above. After incubation in Buffer A, tissues were exposed to a hybridization solution containing both antisense probes at the same time (2 μL of each), allowing combined labeling. After hybridization, sections were washed, counterstained with DAPI, and mounted on slides. The dual-labelling approach enabled the visualization of co-localization or segregation of opsin RNAs within the same anatomical structure, offering a higher functional resolution than single labeling.

#### Imaging of opsin RNAs

Brains were imaged using a Leica SP8 confocal microscope (Leica Microsystem). Parameters (laser power, detector gain) were adjusted for each fluorophore to achieve a good signal-to-noise ratio and to optimize the absence of signal saturation. For each slide, we captured volumetric stacks using a 40x objective (HC PL APO 40x/1.30, Leica Microsystem). The fluorophores were excited at different wavelengths: Quasar 570 (*Lop2* and *Blop*) at 555 nm laser and Quasar 670 (*Lop1* and *Uvop*) at 644 nm (Leica Microsystem). DAPI was excited at 780 nm with an Infra-red tuneable femtosecond laser (InSight X3, Spectra Physics, USA) in two-photon regime.

### Quantification and statistical analyses

Ommatidial types were manually classified by selecting suitable fields of view from double-stained 3D image stacks (Lop1/Blop or Blop/UVop) and tracing individual ommatidia across multiple frames to confirm the identity of the constituent photoreceptor cells. Based on these observations, the frequency of each ommatidial type was determined, along with the ratio of UV- to blue-sensitive photoreceptors.

## References

[1] Wyszecki G., Stiles W.S. (1982).

[2] Gegenfurtner K.R., Kiper D.C. (2003). Color vision. Annu. Rev. Neurosci..

[3] Schiller P.H., Logothetis N.K. (1990). The color-opponent and broad-band channels of the primate visual system. Trends Neurosci..

[4] Kelber A. (2016). Colour in the eye of the beholder: receptor sensitivities and neural circuits underlying colour opponency and colour perception. Curr. Opin. Neurobiol..

[5] Menzel R., Backhaus W., Stavenga D.G., Hardie R.C. (1989). Facets of Vision.

[6] von Frisch K. (1914). Der Farbensinn und Formensinn der Biene. Zool. Jahrb. Abt. Allg. Zool. Physiol. Tiere.

[7] Kühn A. (1924). Zum Nachweis des Farbenunterscheidungsvermögens der Bienen. Naturwissenschaften.

[8] Menzel R., Backhaus W., Gouras P. (1991). Vision and Visual Dysfunction.

[9] Dyer A.G., Paulk A.C., Reser D.H. (2011). Colour processing in complex environments: insights from the visual system of bees. Proc. Biol. Sci..

[10] Avarguès-Weber A., Giurfa M. (2014). Cognitive components of color vision in honey bees: how conditioning variables modulate color learning and discrimination. J. Comp. Physiol. A Neuroethol. Sens. Neural Behav. Physiol..

[11] Avarguès-Weber A., Mota T., Giurfa M. (2012). New vistas on honey bee vision. Apidologie.

[12] Streinzer M., Brockmann A., Nagaraja N., Spaethe J. (2013). Sex and caste-specific variation in compound eye morphology of five honeybee species. PLoS One.

[13] Wakakuwa M., Kurasawa M., Giurfa M., Arikawa K. (2005). Spectral heterogeneity of honeybee ommatidia. Naturwissenschaften.

[14] Wehner R., Bernard G.D., Geiger E. (1975). Twisted and non-twisted rhabdoms and their significance for polarization detection in the bee. J. Comp. Physiol..

[15] Stavenga D.G., de Grip W.J. (1983). Progress in phototransduction. Biophys. Struct. Mech..

[16] Nagata T., Inoue K. (2021). Rhodopsins at a glance. J. Cell Sci..

[17] Autrum H. (1952). Über zeitliches Auflösungsvermögen und Primärvorgänge im Insektenauge. Naturwissenschaften.

[18] Menzel R., Blakers M. (1976). Colour receptors in the bee eye — Morphology and spectral sensitivity. J. Comp. Physiol..

[19] Kien J., Menzel R. (1977). Chromatic properties of interneurons in the optic lobes of the bee. II. Narrow band and colour opponent neurons. J. Comp. Physiol..

[20] Backhaus W. (1991). Color opponent coding in the visual system of the honeybee. Vision Res..

[21] Townson S.M., Chang B.S., Salcedo E., Chadwell L.V., Pierce N.E., Britt S.G. (1998). Honeybee blue- and ultraviolet-sensitive opsins: cloning, heterologous expression in *Drosophila*, and physiological characterization. J. Neurosci..

[22] Chang B.S., Ayers D., Smith W.C., Pierce N.E. (1996). Cloning of the gene encoding honeybee long-wavelength rhodopsin: a new class of insect visual pigments. Gene.

[23] Ribi W.A. (1975). The first optic ganglion of the bee. I. Correlation between visual cell types and their terminals in the lamina and medulla. Cell Tissue Res..

[24] Labhart T. (1980). Specialized photoreceptors at the dorsal rim of the honeybee's compound eye: Polarizational and angular sensitivity. J. Comp. Physiol..

[25] Velarde R.A., Sauer C.D., Walden K.K.O., Fahrbach S.E., Robertson H.M. (2005). Pteropsin: a vertebrate-like non-visual opsin expressed in the honey bee brain. Insect Biochem. Mol. Biol..

[26] Ribi W., Warrant E., Zeil J. (2011). The organization of honeybee ocelli: Regional specializations and rhabdom arrangements. Arthropod Struct. Dev..

[27] Goldsmith T.H., Ruck P.R. (1958). The spectral sensitivities of the dorsal ocelli of cockroaches and honeybees; an electrophysiological study. J. Gen. Physiol..

[28] Ogawa Y., Ribi W., Zeil J., Hemmi J.M. (2017). Regional differences in the preferred e-vector orientation of honeybee ocellar photoreceptors. J. Exp. Biol..

[29] Lichtenstein L., Grübel K., Spaethe J. (2018). Opsin expression patterns coincide with photoreceptor development during pupal development in the honey bee, *Apis mellifera*. BMC Dev. Biol..

[30] Ribi W.A., Engels E., Engels W. (1989). Sex and caste specific eye structures in stingless bees and honey bees (Hymenoptera: Trigonidae, Apidae). Entomol. Gen..

[31] Menzel J.G., Wunderer H., Stavenga D.G. (1991). Functional morphology of the divided compound eye of the honeybee drone (*Apis mellifera*). Tissue Cell.

[32] Lehrer M., Srinivasan M.V., Zhang S.W., Horridge G.A. (1988). Motion cues provide the bee's visual world with a third dimension. Nature.

[33] Srinivasan M.V., Lehrer M., Horridge G.A. (1990). Visual figure-ground discrimination in the honeybee - The role of motion parallax at boundaries. Proc R Soc Ser B-Bio.

[34] Lehrer M., Srinivasan M.V. (1993). Object detection by honeybees: Why do they land on edges?. J. Comp. Physiol..

[35] Lehrer M., Srinivasan M.V., Zhang S.W. (1990). Visual edge-detection in the honeybee and Its chromatic properties. Proc. R. Soc. Lond. B Biol. Sci..

[36] Kirchner W.H., Srinivasan M.V. (1989). Freely flying honeybees use image motion to estimate object distance. Naturwissenschaften.

[37] Srinivasan M.V., Lehrer M., Zhang S.W., Horridge G.A. (1989). How honeybees measure their distance from objects of unknown size. J. Comp. Physiol..

[38] Giurfa M., Vorobyev M., Kevan P., Menzel R. (1996). Detection of coloured stimuli by honeybees: minimum visual angles and receptor specific contrasts. J. Comp. Physiol..

[39] Giurfa M., Vorobyev M., Brandt R., Posner B., Menzel R. (1997). Discrimination of coloured stimuli by honeybees: Alternative use of achromatic and chromatic signals. J. Comp. Physiol. A Neuroethol. Sens. Neural Behav. Physiol..

[40] Giurfa M., Vorobyev M. (1997). The detection and recognition of color stimuli by honeybees: Performance and mechanisms. Isr. J. Plant Sci..

[41] Giurfa M., Zaccardi G., Vorobyev M. (1999). How bees detect coloured targets using different regions of their compound eyes. J. Comp. Physiol. A Neuroethol. Sens. Neural Behav. Physiol..

[42] Kelber A., Somanathan H. (2019). Spatial vision and visually guided behavior in Apidae. Insects.

[43] Srinivasan M.V., Lehrer M., Kirchner W.H., Zhang S.W. (1991). Range perception through apparent image speed in freely flying honeybees. Vis. Neurosci..

[44] Wernet M.F., Perry M.W., Desplan C. (2015). The evolutionary diversity of insect retinal mosaics: common design principles and emerging molecular logic. Trends Genet..

[45] Wehrhahn C. (1979). Sex-specific differences in the chasing behaviour of houseflies (*Musca*). Biol. Cybern..

[46] Perry M.W., Desplan C. (2016). Love spots. Curr. Biol..

[47] Hardie R.C. (1986). The photoreceptor array of the dipteran retina. Trends Neurosci..

[48] Sison-Mangus M.P., Bernard G.D., Lampel J., Briscoe A.D. (2006). Beauty in the eye of the beholder: the two blue opsins of lycaenid butterflies and the opsin gene-driven evolution of sexually dimorphic eyes. J. Exp. Biol..

[49] Bernard G.D., Remington C.L. (1991). Color vision in *Lycaena* butterflies: spectral tuning of receptor arrays in relation to behavioral ecology. Proc. Natl. Acad. Sci. USA.

[50] Olberg R.M. (2012). Visual control of prey-capture flight in dragonflies. Curr. Opin. Neurobiol..

[51] Laughlin S., McGinness S. (1978). The structures of dorsal and ventral regions of a dragonfly retina. Cell Tissue Res..

[52] Labhart T., Nilsson D.E. (1995). The dorsal eye of the dragonfly *Sympetrum* - Specializations for prey detection against the blue sky. J. Comp. Physiol..

[53] Menzel R., Snyder A.W. (1974). Polarised light detection in the bee, *Apis mellifera*. J. Comp. Physiol..

[54] Spaethe J., Briscoe A.D. (2004). Early duplication and functional diversification of the opsin gene family in insects. Mol. Biol. Evol..

[55] Peitsch D., Fietz A., Hertel H., de Souza J., Ventura D.F., Menzel R. (1992). The spectral input systems of hymenopteran insects and their receptor-based colour vision. J. Comp. Physiol..

[56] von Helversen O. (1972). Zur spektralen Unterschiedsempfindlichkeit der Honigbiene. J. Comp. Physiol..

[57] Chittka L., Menzel R. (1992). The evolutionary adaptation of flower colors and the insect pollinators color-vision. J. Comp. Physiol..

[58] Garcia J.E., Hung Y.-S., Greentree A.D., Rosa M.G.P., Endler J.A., Dyer A.G. (2017). Improved color constancy in honey bees enabled by parallel visual projections from dorsal ocelli. Proc. Natl. Acad. Sci. USA.

[59] Neumeyer C. (1981). Chromatic adaptation in the honeybee - Successive color contrast and color constancy. J. Comp. Physiol..

[60] Werner A., Menzel R., Wehrhahn C. (1988). Color constancy in the honeybee. J. Neurosci..

[61] Rister J., Desplan C. (2011). The retinal mosaics of opsin expression in invertebrates and vertebrates. Dev. Neurobiol..

[62] Perry M., Kinoshita M., Saldi G., Huo L., Arikawa K., Desplan C. (2016). Molecular logic behind the three-way stochastic choices that expand butterfly colour vision. Nature.

[63] Wernet M.F., Mazzoni E.O., Çelik A., Duncan D.M., Duncan I., Desplan C. (2006). Stochastic spineless expression creates the retinal mosaic for colour vision. Nature.

[64] Spaethe J., Briscoe A.D. (2005). Molecular characterization and expression of the UV opsin in bumblebees: three ommatidial subtypes in the retina and a new photoreceptor organ in the lamina. J. Exp. Biol..

[65] Qiu X., Vanhoutte K.A.J., Stavenga D.G., Arikawa K. (2002). Ommatidial heterogeneity in the compound eye of the male small white butterfly, *Pieris rapae crucivora*. Cell Tissue Res..

[66] Briscoe A.D. (2008). Reconstructing the ancestral butterfly eye: focus on the opsins. J. Exp. Biol..

[67] Johnston R.J. (2013). Lessons about terminal differentiation from the specification of color-detecting photoreceptors in the *Drosophila* retina. Ann. N. Y. Acad. Sci..

[68] Wernet M.F., Huberman A.D., Desplan C. (2014). So many pieces, one puzzle: cell type specification and visual circuitry in flies and mice. Genes Dev..

[69] Kirschfeld K., Feiler R., Franceschini N. (1978). A photostable pigment within the rhabdomere of fly photoreceptors no. 7. J. Comp. Physiol..

[70] Franceschini N., Kirschfeld K., Minke B. (1981). Fluorescence of photoreceptor cells observed in vivo. Science.

[71] Chou W.H., Hall K.J., Wilson D.B., Wideman C.L., Townson S.M., Chadwell L.V., Britt S.G. (1996). Identification of a novel *Drosophila* opsin reveals specific patterning of the R7 and R8 photoreceptor cells. Neuron.

[72] Friedrich M., Wood E.J., Wu M. (2011). Developmental evolution of the insect retina: insights from standardized numbering of homologous photoreceptors. J. Exp. Zool. B Mol. Dev. Evol..

[73] Ready D.F. (1989). A multifaceted approach to neural development. Trends Neurosci..

[74] Arikawa K. (2003). Spectral organization of the eye of a butterfly, *Papilio*. J. Comp. Physiol. A Neuroethol. Sens. Neural Behav. Physiol..

[75] Arikawa K. (2017). The eyes and vision of butterflies. J. Physiol..

[76] Wakakuwa M., Stavenga D.G., Arikawa K. (2007). Spectral organization of ommatidia in flower-visiting insects. Photochem. Photobiol..

[77] Labhart T., Keller K. (1992). Fine structure and growth of the polarization-sensitive dorsal rim area in the compound eye of larval crickets. Naturwissenschaften.

[78] Dacke M., Nordström P., Scholtz C.H., Warrant E.J. (2002). A specialized dorsal rim area for polarized light detection in the compound eye of the scarab beetle *Pachysoma striatum*. J. Comp. Physiol. A Neuroethol. Sens. Neural Behav. Physiol..

[79] Schmeling F., Wakakuwa M., Tegtmeier J., Kinoshita M., Bockhorst T., Arikawa K., Homberg U. (2014). Opsin expression, physiological characterization and identification of photoreceptor cells in the dorsal rim area and main retina of the desert locust, *Schistocerca gregaria*. J. Exp. Biol..

[80] Blum M., Labhart T. (2000). Photoreceptor visual fields, ommatidial array, and receptor axon projections in the polarisation-sensitive dorsal rim area of the cricket compound eye. J. Comp. Physiol..

[81] Stalleicken J., Labhart T., Mouritsen H. (2006). Physiological characterization of the compound eye in monarch butterflies with focus on the dorsal rim area. J. Comp. Physiol. A Neuroethol. Sens. Neural Behav. Physiol..

